# Genome‐wide analysis reveals the genetic stock structure of hoki (*Macruronus novaezelandiae*)

**DOI:** 10.1111/eva.13317

**Published:** 2021-11-23

**Authors:** Emily Koot, Chen Wu, Igor Ruza, Elena Hilario, Roy Storey, Richard Wells, David Chagné, Maren Wellenreuther

**Affiliations:** ^1^ The New Zealand Institute for Plant and Food Research Ltd Palmerston North New Zealand; ^2^ The New Zealand Institute for Plant and Food Research Ltd Auckland New Zealand; ^3^ The New Zealand Institute for Plant and Food Research Ltd Nelson New Zealand; ^4^ The New Zealand Institute for Plant and Food Research Ltd Te Puke New Zealand; ^5^ Deepwater Group Auckland New Zealand; ^6^ School of Biological Sciences The University of Auckland Auckland New Zealand

**Keywords:** genomics, hoki, *Macruronus*, population, single nucleotide polymorphism, stock structure

## Abstract

The assessment of the genetic structuring of biodiversity is crucial for management and conservation. This is particularly critical for widely distributed and highly mobile deep‐water teleosts, such as hoki (*Macruronus novaezelandiae*). This species is significant to Māori people and supports the largest commercial fishery in New Zealand, but uncertainty about its stock structure presents a challenge for management. Here, we apply a comprehensive genomic analysis to shed light on the demographic structure of this species by (1) assembling the genome, (2) generating a catalogue of genome‐wide SNPs to infer the stock structure and (3) identifying regions of the genome under selection. The final genome assembly used short and long reads and is near complete, representing 93.8% of BUSCO genes, and consisting of 566 contigs totalling 501 Mb. Whole‐genome re‐sequencing of 510 hoki sampled from 14 locations around New Zealand and Australia, at a read depth greater than 10×, produced 227,490 filtered SNPs. Analyses of these SNPs were able to resolve the stock structure of hoki into two genetically and geographically distinct clusters, one including the Australian and the other one all New Zealand locations, indicating genetic exchange between these regions is limited. Location differences within New Zealand samples were much more subtle (global *F*_ST_  = 0.0006), and while small and significant differences could be detected, they did not conclusively identify additional substructures. Ten putative adaptive SNPs were detected within the New Zealand samples, but these also did not geographically partition the dataset further. Contemporary and historical *N*_e_ estimation suggest the current New Zealand population of hoki is large yet declining. Overall, our study provides the first genomic resources for hoki and provides detailed insights into the fine‐scale population genetic structure to inform the management of this species.

## 1 INTRODUCTION

Defining biologically meaningful units for sustaining biodiversity is one of the major goals of population management and conservation biology (Allendorf et al., 2010b; Moritz, 1994). In particular, the detection of genetic structure provides a crucial tool to identify isolated units, and to assess the degree of connectivity among populations (Bernatchez, 2016). Neglecting consideration of population structure may increase risks of overexploitation or mismanagement (Waples, 1998). Marine ecosystems are traditionally considered to be highly connected, and this is typically attributed to the large population sizes of many marine species, coupled with the presence of few barriers to gene flow (Nielsen et al., 2009). In addition, in many marine teleost species, the early life history is characterized by the presence of a planktonic larval stage during which larvae can be transported over long distances by ocean currents (e.g. 100s of km). Based on these characteristics, it is expected that marine species are usually highly connected owing to the combination of large population sizes and high dispersal, which makes it more challenging to characterize population structures accurately.

Small datasets containing neutral loci have been widely used to analyse population structure, gene flow and demographic changes over time. However, the small numbers of markers often lacked the statistical power to detect low rates of genetic differentiation in the high gene flow environments common for marine teleost species (Nielsen et al., 2009). Recent population genomic approaches employing thousands of genome‐wide markers hold promise to provide the degree of resolution required for essentially any socioeconomic or ecologically important marine species (Ellegren, 2014). While the field is still evolving, numerous studies on marine species already demonstrate its potential to offer deeper insight into the dynamics of natural populations (Hohenlohe et al., 2010; Larson et al., 2014).

The ability to apply large numbers of DNA markers to conduct dense genome scans not only has greatly enhanced the power to identify genomic regions exhibiting genetic structure (Nielsen et al., 2012), but also has enabled identification of outlier regions associated with adaptation. Adaptive genomic signatures may be associated with local adaptation or reveal traces of cryptic population structure obscured by gene flow across most of the genome (Duranton et al., 2018; Gagnaire et al., 2015). These outlier loci can reveal regions in the genome of genetic differentiation where neutral markers often remain uninformative, and can prove useful to delineate locally adapted stocks and redefine conservation units (Funk et al., 2012). This approach is appealing because selection may be more efficient than genetic drift in opposing the homogenizing effect of migration, in particular when populations have large effective population sizes, which is the case for many important fisheries species. Genome scans work by detecting significant departures from genomic background patterns observed (Ahrens et al., 2018), while Gene‐Environment‐Associations (GEA) methods work by identifying genetic variants associated with particular environmental factors (Dallaire et al., 2021). However, outlier loci can also arise through a wide variety of evolutionary mechanisms apart from local adaptation (Bierne et al., 2011), in particular in response to varying patterns of recombination (Booker et al., 2020). Such patterns are commonly caused by structural genomic variants (Mérot et al., 2020; Wellenreuther & Bernatchez, 2018; Wellenreuther et al., 2019), and recent work indicates that these can affect more base pairs than SNP variants (Catanach et al., 2019), and are widespread throughout the genome.

**FIGURE 1:**
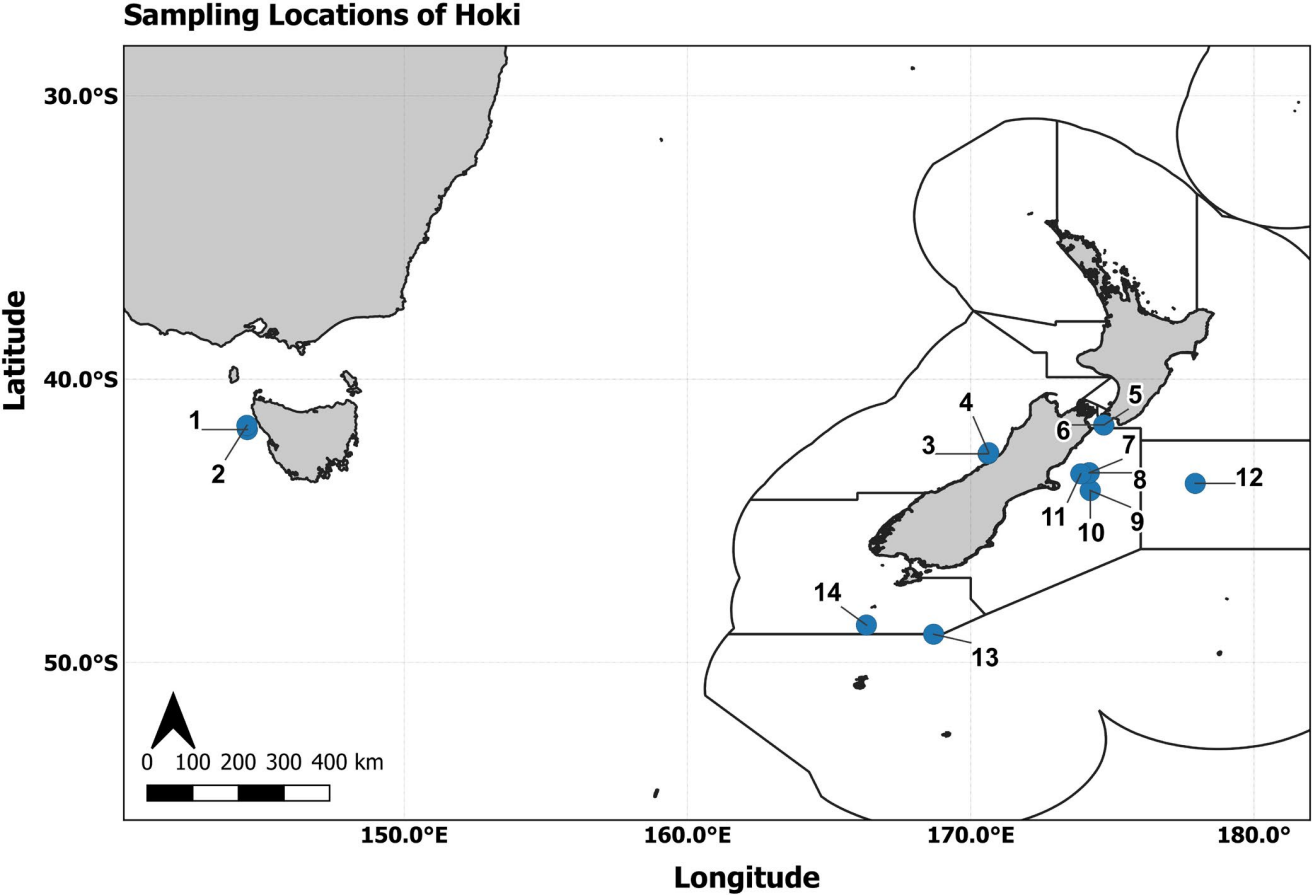
Sampling locations of hoki in New Zealand and Australia used for the population genomic part of this study. See Table 1 for site information

Here, we assess the population genomic structure of hoki (*Macruronus novaezelandiae*, Family: Merlucciidae), which supports one of the most valuable deep‐water fisheries in New Zealand. Hoki are widely distributed throughout New Zealand and Australian waters, being found in most abundant quantities in depths of 200–800 m (Horn & Sullivan, 1996; Livingston & Schofield, 1996). They have long pelagic larval and juvenile phases, maturing at the age of four, and exhibit extensive migratory behaviours (Horn, 2011). The current stock assessment for hoki in New Zealand is based on an assumed two‐stock migration model between the Western and Eastern stocks (Figure 1). These have been defined based on data showing that fish in different geographical locations grow and mature at different rates and have different morphometric characters (Horn & Sullivan, 1996; Livingston & Schofield, 1996; Livingston & Sullivan, 2007). The Western stock encompasses spawning hoki from the West Coast of the South Island of New Zealand. The larvae and juveniles originating from this stock are thought to be then transported to the East Coast nurseries feeding areas on the Western Chatham Rise via the Westland and D'Urville currents (Smith et al., 1996). As young adults, these fish are then thought to migrate from the Chatham Rise to feeding grounds in sub‐Antarctic waters, and subsequently moving between these and the spawning grounds on the West Coast of the South Island as mature adults. In contrast, hoki belonging to the Eastern stock are thought to spawn in the Cook Strait (and at other locations east of the South Island), with larvae and juveniles then migrating from these locations to the Chatham Rise nursery and feeding grounds. Juveniles are assumed to recruit to their respective stocks at maturity at ages of 3–8 years (O'Driscoll, 2004), and mature adults are thought to move between the Chatham Rise and the known spawning grounds in Cook Strait and at Pegasus Canyon. In the spawning grounds, hoki typically form large midwater aggregations, consisting almost entirely of the species.

Modern genomic technologies offer a powerful toolset to independently determine stock structure in this species, but has not been used to date. To enable assessment of this species using genomics solutions and given the significance of this species to Māori, Te Ohu Kaimoana and kaitiaki (cultural guardians) provided advice on a process to manage the gathering, storage, access and use of genetic data. We assembled a high‐quality de novo genome using a combination of short‐ and long‐read sequencing. To investigate the genomic stock structures, we sampled 12 locations in New Zealand and two locations in Australia (≥30 individuals per location) and performed whole genome sequencing at greater than 10× coverage to generate a powerful genome‐wide SNP dataset. This genome‐wide dataset was then used to assess the degrees of genetic diversity between all sampling sites to identify independent clusters, and how these are related to other sampling locations. The dataset was further used to test for population genetic differentiation using both neutral SNPs and putative adaptive SNPs. Our results are compared and discussed in the light of previous studies on hoki, and other teleost species.

## 2 MATERIALS AND METHODS

### 2.1 Indigenous considerations

Gathering genomic data requires strong relationships with indigenous peoples (Hudson et al., 2020). Engagement with Te Ohu Kaimoana started before the operational aspects of the project were initiated and carried on throughout the project. Specifically, a working group was developed to advise on a process to manage the gathering, storage, access and use of samples and genetic data before, during and after the research presented here. An agreement considering Māori tikanga (traditional protocols) was set up for the genomic data lifecycle, and the raw and analysed data were placed in a managed repository based in New Zealand.

### 2.2 Sample and metadata collection and DNA extraction

A single adult female hoki from the Cook Strait was sampled in May 2020 (commercial fishing vessel FV Otakou) to generate DNA for the genome assembly. Five tissues were preserved in RNAlater following the manufacturer's instructions: brain, gills, liver, heart and white muscle. High‐quality DNA was extracted from preserved liver with a CTAB‐based extraction buffer as follows: 100 mg of tissue was homogenized in 1 ml CTAB buffer (2% CTAB [hexadecyltrimethyl ammonium bromide], 2% PVP K40 [polyvinyl pyrrolidinone K40], 2 M NaCl, 25 mM EDTA, 100 mM Tris‐HCl pH 8.0) with a sterile plastic pestle, on ice. After adding 25 µl Proteinase K (20 mg/ml) and 10 µl 1 M dithiothreitol, the mixture was incubated at 50°C for 18 h in a thermomixer 30 s on, 300 rpm, 30 min off. The sample was extracted once with an equal volume of chloroform:isoamyl alcohol (24:1 v/v), by gently mixing by hand for 5 min. The phases were separated by centrifugation (5 min at 16,089 *g* at room temperature), and the aqueous phase was transferred to a new tube and precipitated with 2 volumes of 100% ethanol at room temperature. After ~5 min, the DNA was collected by centrifugation (10 min at 13,000 rpm at room temperature). The pellet was dissolved in 400 µl TE buffer (10 mM Tris‐HCl, 1 mM EDTA pH 7.5) and 100 µl 5 M NaCl. RNA was digested by adding 8 µl of RNase A (50 mg/ml) and incubating at room temperature for 5 min. Twenty‐five microlitres of 10% SDS was added, and the sample was incubated in the thermomixer at 37°C at 300 rpm for 15 min. The sample was extracted with an equal volume of chloroform:isoamyl alcohol (24:1 v/v), mixing gently by hand for 5 min, and the phases were separated as described above. The DNA contained in the aqueous phase was precipitated with 0.7 volumes isopropanol, mixed, and incubated at room temperature for 10 min. The DNA was collected by centrifugation (10 min at 13,000 rpm at room temperature). The pellet was washed with 1 ml 70% ethanol and centrifuged again. The almost invisible pellet was dissolved in 75 µl TE buffer by letting it resuspend overnight at 4°C. The quality of the DNA was assessed by absorbance (A260/280 = 1.91, A260/230 = 2.35) and gel electrophoresis (average size 40 kbp) and quantified with a high sensitivity fluorescent‐based method.

For the population genomics analysis, individual fish were sampled from commercial fishing vessels in New Zealand and Australian waters between May and September 2020 (Figure 1). In total, two sets of 100 individual samples were collected per trawl and data were recorded on fish size and sex. An individual larger than 75 cm was considered to be an adult, and smaller individuals were classed as juveniles but sufficiently old to have separated as per the stock model hypothesis. As fish under 45 cm were still deemed to be at risk of being from either presumptive stock, these were not sampled. Some adults were classed as spawning individuals when they were collected during July and August at known spawning sites, and these were as follows: Hokitika Canyon, Kahurangi (both these are off the West Coast of the South Island of New Zealand) and Cook Strait and Pegasus Canyon (the latter off the East Coast of the South Island). The GPS location of trawls was recorded and used as location indicators. One fin clip was collected from each fish and stored in 95% ethanol in a screw‐cap tube at room temperature until DNA extraction. A random subset of 30 or 40 fish was used from 14 collection sites for the DNA extraction and sequencing. The collection sites were selected to represent a geographic distribution, including various life stages. A description of the 510 hoki samples can be seen in Table 1. DNA extraction was performed at Neogen GeneSeek, Lincoln, NE, USA, based on the LGC sbeadex™ magnetic bead kit that uses a two‐step binding mechanism to provide high‐quality DNA for downstream SNP and NGS protocols (www.lgcgroup.com).

**TABLE 1:** Description of the hoki sampling

Sample site map ID	Sampling site name	Location	Stage	Region	Latitude	Longitude	Vessel	Time of landing	Number of individual fish sequenced
1	Tasmania A1	Tasmania	Adult	Tasmania	41 47.014 S	144 28.008 E	Tokatu	Jul‐20	30
2	Tasmania A2	Tasmania	Adult	Tasmania	41 38.057 S	144 27.019 E	Tokatu	Jul‐20	30
3	West Coast 1	Hokitika canyon	Spawning fish	West coast south island	42 38.32 S	170 36.87 E	Thomas Harrison	Jul‐20	40
4	West Coast 2	Hokitika canyon	Spawning fish	West coast south island	42 36 S	170 38 E	Thomas Harrison	Jul‐20	40
5	Cook Strait 1	Cook Strait St Bank	Spawning fish	Cook Strait	41 37.4 S	174 41.8 E	Otakou	Jul‐20	40
6	Cook Strait 2	Cook Strait St Bank	Spawning fish	Cook Strait	41 37.1 S	174 41.7 E	Otakou	Jul‐20	40
7	FMA3 J1	West Mernoo	Juvenile	FMA3	43 18.4 S	174 10.9 E	San Discovery	Aug‐20	40
8	FMA3 A1	West Mernoo	Adult	FMA3	43 18.4 S	174 10.9 E	San Discovery	Aug‐20	40
9	FMA3 J2	West Mernoo	Juvenile	FMA3	43 56.0 S	174 13.2 E	San Discovery	Aug‐20	40
10	FMA3 A2	West Mernoo	Adult	FMA3	43 56.0 S	174 13.2 E	San Discovery	Aug‐20	40
11	FMA3 A3	Pegasus Canyon	Adult	FMA3	43 20.7 S	173 53.1 E	San Enterprise	Jul‐20	30
12	FMA4	North Rise	Adult	FMA4	43 40.986 S	177 55.488 E	San Enterprise	Jun‐20	40
13	FMA6 Snare	Snares Shelf	Adult	FMA6	49 0.29 S	168 41.4 E	Amaltal Enterprise	Jul‐20	30
14	FMA6 Norgie	Norgie hole	Adult	FMA6	48 40.9 S	166 19.1 E	Amaltal Enterprise	Jul‐20	30

### 2.3 Genome sequencing and assembly

High molecular weight DNA was sent in July 2020 to the Australian Genome Research Facility (AGRF) for short‐ and long‐read sequencing. The DNA was quality checked by AGRF upon arrival, and passed quality thresholds. The TrueSeq DNA Nano kit (Illumina) was used for library preparation for the Illumina sequencing, and the Illumina NovaSeq 6000 was used for short‐read sequencing (150 bp PE reads). For long‐read sequencing, the Oxford Nanopore Technologies (ONT) technology was used. Three MinION FLO‐MIN106 flow cells were run in‐house and at AGRF, to verify that DNA could be sequenced with this technology, which produced approximately 3 Gb of sequencing data. Then, ONT libraries were prepared at AGRF using the SQK‐LSK109 kit and run using a PromethION FLO‐PRO002 flow cell, using minKNOW version 4.0.5 and base calling using Guppy 4.0.11, producing 7.57 M reads totalling 19.7 Gb of data (passed reads).

Kmer analysis was performed using jellyfish v2.2.10 with kmer size of 21. The ONT reads generated in‐house (MinION) were base‐called using Guppy v4.2.2 with parameters ‘‐‐compress_fastq ‐‐input_path ‐‐save_path ‐‐flowcell FLO‐MIN106 –kit SQK‐LSK109 ‐x “cuda:0”’ and quality‐assessed using pycoqc v2.5.0.21. The long‐read assembly was generated using FLYE v2.8.1 with default parameters based on long reads generated from both in‐house (MinION) and at AGRF (MinION and PromethION). It was then subjected to two rounds of short‐read error‐correction using PILON v1.23 with parameters ‘‐‐genome FLYE.fasta ‐‐frags mapped.bam ‐‐output ‐‐outdir ‐‐changes ‐‐diploid ‐‐fix all ‐‐threads 60 ‐‐flank 5’. Assembly completeness was assessed using BUSCO v5.1.2 with parameters ‘‐l actinopterygii_odb10 ‐o test_long ‐m geno ‐‐augustus ‐‐augustus_species zebrafish ‐c 20 ‐‐long ‐‐out_path busco/’ (Simão et al., 2015).

### 2.4 Whole genome sequencing, variant calling, and filtering

Illumina paired‐end (PE) short reads were generated at Neogen GeneSeek. In total, 510 fish samples were sequenced with a target read depth greater than 10×. A summary of the sequencing data is in Table S1. Variant calling was performed using the hoki genome assembled from the ONT data as a reference. Illumina PE reads were mapped to the reference genome using BWA‐mem (v0.7.17), and the resulting sam files were converted to bam files using Samtools v1.9 (Li et al., 2009). SNP calling was performed using Freebayes v1.1.0 (Garrison & Marth, 2012) with the following parameters: ‐p 2 –C3 –m 10 ‐‐min‐coverage 15 and ‐‐max‐coverage 500. Variant filtering was performed using vcftools v0.1.14 (Danecek et al., 2011), applying the following filters: ‐‐max‐alleles = 2, ‐‐max‐missing = 0.95, ‐‐maf = 0.02, ‐‐remove‐indels.

### 2.5 Outlier scans for stock structure assessment

Outlier SNPs were identified using three methods – tess3 (Caye et al., 2016), pcadapt (Luu et al., 2017) and LEA (Frichot & François, 2015). Tess3 searches for genomic variants under selection by applying matrix factorization algorithms to allele frequencies; pcadapt runs genome‐wide selection scans based on principal component analyses; and LEA calculates *F*_ST_ statistics from ancestral allele frequencies that are estimated using the packages snmf function. Tess3 was implemented using the tess3r package in R v3.6.1 applying the following parameters: method = “projected.ls”, ploidy = 2, max.iteration = 5000, rep = 10, keep = “best”, tolerance = 1e−05. The R package pcadapt v4.3.3 was implemented using default settings and method = “mahalanobis”. The following parameters were used to run LEA's snmf function: genotype, *K* = 1:14, entropy = T, ploidy = 2, repetitions = 10, tolerance = 1e−05. The three analyses were conducted on two different datasets – the first contained all filtered SNPs and all hoki samples (510 samples); the second contained all filtered SNPs but only New Zealand samples (450 samples). A *Q*‐value of 0.05 was used as a threshold of statistical significance for all three analyses. *Q*‐values were calculated using the qvalue R package (Dabney et al., 2010). Variants that met this threshold for all three analyses were considered as outliers that were putatively under divergent selection, whilst any variant that was not considered under selection by any of the analyses was considered to be putatively neutral.

### 2.6 Stock structure and size

Genetic diversity and population structure were investigated using six datasets: (1) all SNPs and all hoki samples, (2) all SNPs and New Zealand‐only samples, (3) neutral SNPs and all hoki samples, (4) neutral SNPs and New Zealand‐only samples, (5) adaptive SNPs and all hoki samples, and (6) adaptive SNPs and New Zealand‐only samples. Nucleotide diversity (*π*), observed (*H*_o_) and expected (*H*_e_) heterozygosity and Tajima's *D* statistics were calculated for each sampling site and for each of the six datasets using vcftools v0.1.14. Weir and Cockerham's (1984) pairwise *F*_ST_ distances and accompanying *p*‐values were also estimated for each dataset and each sampling site using R package StAMPP (Pembleton et al., 2013), applying: nboots = 1000, per cent = 95. Population structure was investigated using the adegenet R packages find.clusters and DAPC functions (Jombart & Ahmed, 2011). Find.clusters was used to initially explore the optimal *K* value (number of ancestral clusters) for each dataset before the DAPC analysis was run using this optimized *K* value, in addition to an optimized number of principal components (PCs). The snmf function in the R package LEA was used to explore the ancestral admixture (Frichot & François, 2015). A range of *K* values were explored (*K* = 1:14), in addition to the following parameter settings: entropy = T, ploidy = 2, repetitions = 10, tolerance = 0.00001. For the DAPC and LEA analyses, individual analyses were run for each of the six datasets. Contemporary effective population size (*N*_e_) was estimated for New Zealand samples using the linkage disequilibrium (LD) method in NeEstimator v2 (Do et al., 2014), whilst SNeP v1.1 was used to estimate historical *N*_e_ (Barbato et al., 2015). The complete SNP dataset for New Zealand samples was thinned by 10,000 sites in vcftools before contemporary and historical *N*_e_ values were estimated.

## 3 RESULTS

### 3.1 Reference genome assembly

In total, 22.7 Gb of ONT long‐read sequencing data was generated. PromethION and MinION sequencing resulted in 19.69 and 3.01 Gb of passed reads with N50 greater than 4.9 and 5.2 kb, respectively. Assembly using the ONT data and using the FLYE software resulted in a total of 501,488,236 bp assembled into 566 scaffolds (Table 2). The N50 of scaffolds was greater than 11 Mb and 15 scaffolds accounted for more than half the total assembly (L50). The largest scaffolds were approximately 26 Mb in length. BUSCO analysis after polishing using Illumina short reads and using PILON resulted in 93.8% complete BUSCOs (0.9% duplicated) out of 3640 zebrafish conserved genes.

**TABLE 2:** Assembly metrics for the hoki reference genome

Number of scaffolds	566
Total size of scaffolds	501,488,236 bp
Longest scaffold	25,972,667 bp
Mean scaffold size	886,022 bp
N50 scaffold length	11,052,189 bp
L50 scaffold count	15
Complete BUSCOs (single)	92.90%
Complete BUSCOs (duplicated)	0.90%
Fragmented BUSCOs	1%
Missing BUSCOs	5.20%

### 3.2 Individual hoki samples sequencing, variant calling and filtering

A total of 6148 Gb of Illumina sequencing data were generated for all 510 sampled fish (Table S1), corresponding to a mean of 12.05 Gb of data per individual (median 11.68 Gb), which corresponds to a mean read depth of ~24× based on the assembled 501 Mb hoki genome. Only four individuals had less than 9 Gb of total data and the individual with the lowest yield had greater than 5 Gb of data, which is still greater than the target 10× coverage. The mapping rate to the reference genome ranged between 81.5% and 94.9%, with a median of 93%, and only seven individuals had mapping rates lower than 90%. In total, 3,906,729 raw variants were detected of which 227,490 were retained after filtering.

### 3.3 Outlier scans

Tess3 identified 8268 SNPs under selection in the dataset containing all samples, and 2862 in the dataset consisting of only New Zealand samples (Table 3; Figure S1). Pcadapt identified 3502 SNPs and 3720 in each dataset, respectively. LEA identified 9778 SNPs and 5396 in each dataset, respectively. 2797 SNPs were identified as outliers across all three analyses for the complete dataset, whilst only 10 SNPs were identified across all analyses for the New Zealand‐only dataset (A list of outlier SNPs is available upon request). This subsequently resulted in the two neutral datasets having 216,572 SNPs (all hoki) and 216,974 SNPs (New Zealand only) each.

**TABLE 3:** Outlier SNPs

**New Zealand and Australia**	**Threshold**	**<1e−04**	**<0.001**	**<0.01**	**<0.025**	**<0.05**	**<0.1**
tess3	*p*‐Value	4555	7227	13,203	18,772	25,985	37,936
	*q*‐Value	2045	3221	5364	6651	8268	10,502
	Local FDR	1467	2230	3662	4516	5323	6401
pcadapt	*p*‐Value	2191	3264	5855	8283	11,373	17,065
	*q*‐Value	1286	1698	2404	2929	3502	4328
	Local FDR	1043	1275	1722	1975	2304	2733
LEA	*p*‐Value	5687	8274	14,656	19,908	26,346	36,523
	*q*‐Value	3506	4616	6708	8094	9778	12,321
	Local FDR	2727	3508	4739	5565	6383	7498
						2797	

**New Zealand only**	**Threshold**	**<1e−04**	**<0.001**	**<0.01**	**<0.025**	**<0.05**	**<0.1**
tess3	*p*‐Value	909	3696	11,153	17,031	24,175	35,554
	*q*‐Value	4	11	125	921	2862	6068
	Local FDR	0	9	60	436	1411	3299
pcadapt	*p*‐Value	1419	3173	8000	11,546	15,578	21,778
	*q*‐Value	399	718	1496	2369	3720	5985
	Local FDR	284	459	921	1313	1947	3219
LEA	*p*‐Value	2130	5060	12,489	18,347	24,782	35,077
	*q*‐Value	140	554	2058	3559	5396	8421
	Local FDR	101	346	1132	1998	3030	4589
						10	

Note

Results of tess3, pcadapt and LEA for all sampling sites (top table) and New Zealand sites (bottom table). SNPs meeting the 0.05 *q*‐value threshold are highlighted in grey, with the total number of intercepting SNPs noted at the bottom of this column.

### 3.4 Population structure analyses

#### 3.4.1 All SNPs and all hoki samples

**FIGURE 2:**
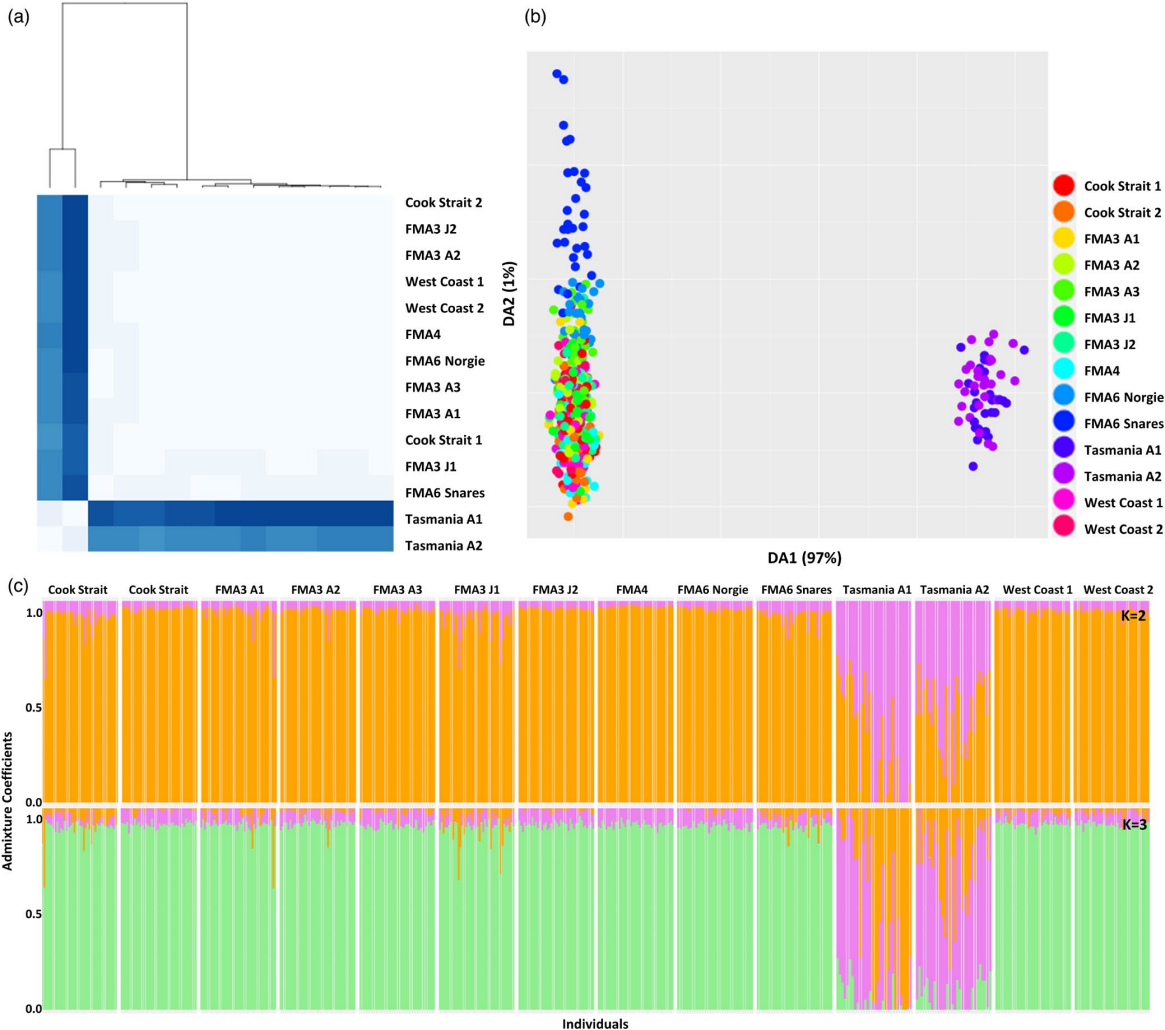
Population genomic analysis outputs for the hoki dataset consisting of all SNPs and all sampling sites: (a) pairwise *F*_ST_ heatmap with hierarchical clustering dendrogram—darker blues indicate higher pairwise *F*_ST_ values and lighter blues indicate lower pairwise *F*_ST_ values, (b) DAPC scatterplot of DA1 (97%) and DA2 (1%) with points coloured by sampling sites, and (c) LEA ancestral admixture plots for *K* = 2 and *K* = 3

Mean nucleotide diversity across New Zealand sampling sites ranged from 0.163 to 0.169 (Table 4). Mean nucleotide diversity for the two Australian sites was slightly higher (0.189 and 0.203). Mean observed heterozygosity for New Zealand sites ranged from 0.173 to 0.188 and was greater than expected heterozygosity in all instances. Mean observed heterozygosity for Australian sites was higher (0.234 and 0.260) and also higher than expected heterozygosity. Mean Tajima's *D* was negative across all New Zealand sites and one Australian site (Tasmania A2); however, it was positive for the other (Tasmania A1) site. Pairwise *F*_ST_ was examined between all sampling sites, *F*_ST_ estimates were very low among New Zealand sites, ranging from 0.00027 (*p* < 0.001) to 0.00164 (*p* < 0.001) (mean = 0.00067), with pairwise *F*_ST_ between the two Australian sites being higher (0.0025, *p* < 0.001) (Table 5). Pairwise *F*_ST_ distance between all New Zealand and all Australian sites was 0.02139 (*p* < 0.001), and when the *F*_ST_ genetic distances were examined as a heatmap, clear genetic differentiation between New Zealand and Australian samples was visible (Figure 2a).

**TABLE 4:** Diversity statistics for three of the datasets: all SNPs and all sampling sites; neutral SNPs and New Zealand sites only; and adaptive SNPs and New Zealand sites only

	*π* (mean)	*H*_o_ (mean)	*H*_e_ (mean)	Tajima's *D* (mean)
All SNPs, all sampling sites				
Cook Strait 1	0.169	0.188^a^	0.167	−0.342
Cook Strait 2	0.164	0.176^a^	0.162	−0.386
FMA3 A1	0.167	0.182^a^	0.165	−0.359
FMA3 A2	0.165	0.178^a^	0.163	−0.379
FMA3 A3	0.165	0.178^a^	0.162	−0.442
FMA3 J1	0.169	0.188^a^	0.167	−0.331
FMA3 J2	0.164	0.176^a^	0.162	−0.385
FMA4	0.163	0.173^a^	0.161	−0.397
FMA6 Norgie	0.164	0.175^a^	0.161	−0.449
FMA6 Snares	0.168	0.185^a^	0.165	−0.405
Tasmania A1	0.203	0.260^a^	0.199	0.012
Tasmania A2	0.189	0.234^a^	0.186	−0.140
West Coast 1	0.166	0.180^a^	0.164	−0.375
West Coast 2	0.166	0.179^a^	0.163	−0.377
Neutral SNPs, New Zealand only				
Cook Strait 1	0.171	0.191^a^	0.169	−0.317
Cook Strait 2	0.167	0.179^a^	0.165	−0.360
FMA3 A1	0.170	0.185^a^	0.167	−0.334
FMA3 A2	0.167	0.181^a^	0.165	−0.353
FMA3 A3	0.168	0.180^a^	0.165	−0.417
FMA3 J1	0.171	0.191^a^	0.169	−0.307
FMA3 J2	0.167	0.179^a^	0.165	−0.359
FMA4	0.166	0.176^a^	0.164	−0.371
FMA6 Norgie	0.167	0.178^a^	0.164	−0.424
FMA6 Snares	0.171	0.188^a^	0.168	−0.382
West Coast 1	0.168	0.183^a^	0.166	−0.348
West Coast 2	0.168	0.182^a^	0.166	−0.350
Adaptive SNPs, New Zealand only				
Cook Strait 1	0.155	0.150	0.159	−0.281
Cook Strait 2	0.092	0.098^a^	0.091	−0.653
FMA3 A1	0.192	0.200^a^	0.189	−0.060
FMA3 A2	0.107	0.115^a^	0.106	−0.565
FMA3 A3	0.146	0.130	0.143	−0.411
FMA3 J1	0.175	0.180^a^	0.173	−0.161
FMA3 J2	0.114	0.110	0.112	−0.524
FMA4	0.100	0.105^a^	0.099	−0.608
FMA6 Norgie	0.131	0.110	0.129	−0.497
FMA6 Snares	0.138	0.143^a^	0.135	−0.460
West Coast 1	0.166	0.178^a^	0.164	−0.213
West Coast 2	0.115	0.123^a^	0.114	−0.516

Note

*H*_e_, expected heterozygosity; *H*_o_, observed heterozygosity; *π*, nucleotide diversity.

^a^ Observed heterozygosity is higher than expected heterozygosity.

**TABLE 5:** Pairwise *F*_ST_ for neutral (top matrix) and adaptive (bottom matrix) SNPs for all sampling sites

	Cook Strait 1	Cook Strait 2	FMA3 A1	FMA3 A2	FMA3 A3	FMA3 J1	FMA3 J2	FMA4	FMA6 Norgie	FMA6 Snares	Tasmania A1	Tasmania A2	West Coast 1	West Coast 2
Cook Strait 1		0.0004***	0.0005***	0.0004***	0.0007***	0.0009***	0.0005***	0.0006***	0.0008***	0.0013***	0.0117***	0.0085***	0.0005***	0.0004***
Cook Strait 2	0.0005		0.0004***	0.0004***	0.0005***	0.00010***	0.0003***	0.0004***	0.0007***	0.0013***	0.0131***	0.0094***	0.0003***	0.0003***
FMA3 A1	0.0006	0.0000		0.0004***	0.0007***	0.0010***	0.0004***	0.0005***	0.0007***	0.0012***	0.0124***	0.0089***	0.0003***	0.0003***
FMA3 A2	0.0004	0.0000	0.0000		0.0004***	0.0010***	0.0003***	0.0004***	0.0006***	0.0011***	0.0129***	0.0093***	0.0005***	0.0004***
FMA3 A3	0.0013*	0.0008	0.0012*	0.0014*		0.0010***	0.0004***	0.0005***	0.0004***	0.0009***	0.0128***	0.0093***	0.0006***	0.0005***
FMA3 J1	0.0012**	0.0012**	0.0013***	0.0012**	0.0016**		0.0010***	0.0010***	0.0012***	0.0016***	0.0122***	0.0091***	0.0010***	0.0009***
FMA J2	0.0015***	0.0008*	0.0001	0.0004	0.0022***	0.0016***		0.0003***	0.0005***	0.0012***	0.0131***	0.0095***	0.0004***	0.0004***
FMA4	0.0010**	0.0000	0.0002	0.0000	0.0016**	0.0012**	0.0004		0.0006***	0.0013***	0.0134***	0.0096***	0.0004***	0.0004***
FMA6 Norgie	0.0009	0.0004	0.0008	0.0000	0.0003	0.0015**	0.0009	0.0000		0.0009***	0.0130***	0.0093***	0.0008***	0.0006***
FMA6 Snares	0.0012*	0.0015**	0.0010	0.0009	0.0020***	0.0013**	0.0012*	0.0017***	0.0008		0.0124***	0.0094***	0.0012***	0.0011***
Tasmania A1	0.2395***	0.2511***	0.2452***	0.2487***	0.2300***	0.2394***	0.2490***	0.2510***	0.2334***	0.2269***		0.0024***	0.0128***	0.0128***
Tasmania A2	0.2152***	0.2272***	0.2213***	0.2242***	0.2075***	0.2155***	0.2252***	0.2266***	0.2110***	0.2039***	0.0027**		0.0093***	0.0093***
West Coast 1	0.0002	0.0000	0.0000	0.0002	0.0016**	0.0014***	0.0010**	0.0005	0.0001	0.0024***	0.2460***	0.2219***		0.0003***
West Coast 2	0.0004	0.0002	0.0002	0.0000	0.0013**	0.0012***	0.0008*	0.0000	0.0000	0.0007	0.24567***	0.2215***	0.0000	

*p < 0.05, **p < 0.01, ***p < 0.001.

Kmeans clustering analysis (using find.clusters) of all hoki samples and all SNPs identified two clusters (*K* = 2) within the dataset (Figure S2a,b). When further examined via DAPC analysis, the two clusters clearly identified two geographically and genetically distinct groups – one containing all New Zealand hoki samples and one containing all Australian hoki samples (Figure 2b). This was further supported by the LEA ancestral admixture analysis, which also identified *K* = 2 as the optimal number of clusters (Figure S2c), and also identified the New Zealand and Australian samples as belonging to two separate clusters (Figure 2c).

The complete SNP dataset was also examined with only New Zealand samples included to determine whether there was any additional structure among the New Zealand sampling sites. The kmeans clustering analysis determined there was one cluster (*K* = 1) within the dataset (Figure S3a,b), with the DAPC scatterplot displaying overlap between all sampling sites (Figure S4). FMA6 (FMA denotes Fisheries Management Areas) Snares and FMA3 J1 did, however, display a low rate of genetic divergence from the other sites when DA 1 and DA 2 were examined. However, it should be noted here that a replicate trawl from FMA3 did not show the same pattern as FMA3 J1. The LEA ancestral admixture analysis also determined that the optimal number of ancestral populations for the New Zealand samples was *K* = 1 (Figure S3c).

#### 3.4.2 Neutral SNPs for all hoki and New Zealand only

**FIGURE 3:**
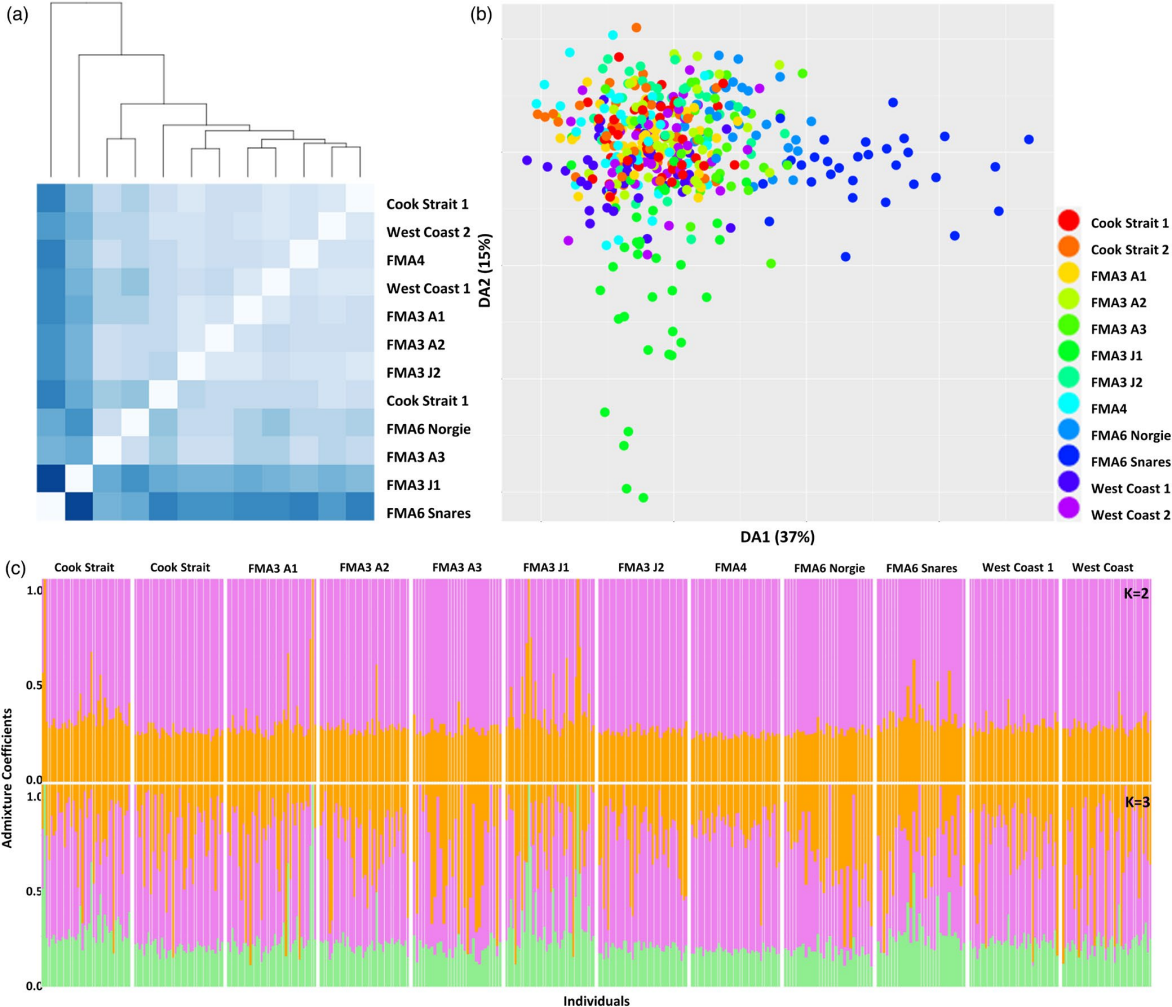
Population genomic analysis outputs for the hoki dataset consisting of neutral SNPs and New Zealand sampling sites only: (a) pairwise *F*_ST_ heatmap with hierarchical clustering dendrogram—darker blues indicate higher pairwise *F*_ST_ values and lighter blues indicate lower pairwise *F*_ST_ values, (b) DAPC scatterplot of DA1 (37%) and DA2 (15%) with points coloured by sampling site, and (c) LEA ancestral admixture plots for *K* = 2 and *K* = 3

Mean nucleotide diversity across neutral SNPs and all hoki ranged from 0.166 to 0.171 within New Zealand sites, and slightly higher for the two Australian sites (0.181–0.195) (Table S2). Mean observed heterozygosity for the New Zealand sites ranged from 0.176 to 0.191, and from 0.225 to 0.249 for the two Australian sites. Observed heterozygosity was higher than expected heterozygosity in all instances. Mean Tajima's *D* was negative for all sites and ranged from −0.424 to −0.307 for the New Zealand sites, and from −0.232 to −0.095 for the two Australian sites. Pairwise *F*_ST_ distance between all New Zealand and all Australian sites was 0.01026 (*p* < 0.001). Pairwise *F*_ST_ distance among New Zealand sites ranged from 0.00026 (*p* < 0.001) to 0.00163 (*p* < 0.001) (mean = 0.00067), and pairwise *F*_ST_ for the two Australian sites was 0.0024 (*p* < 0.001) (Table 5). Mean nucleotide diversity, heterozygosity and Tajima's *D* results for the New Zealand‐only neutral SNPs were the same as those for the all‐hoki neutral dataset (Table 4); however, *F*_ST_ distance among New Zealand sites was slightly lower, ranging from 0.00026 (*p* < 0.001) to 0.00158 (*p* < 0.001) (mean = 0.00062) (Table 6; Figure 3a).

**TABLE 6:** Pairwise *F*_ST_ for neutral (top matrix) and adaptive (bottom matrix) SNPs for New Zealand sampling sites

	Cook Strait 1	Cook Strait 2	FMA3 A1	FMA3 A2	FMA3 A3	FMA3 J1	FMA3 J2	FMA4	FMA6 Norgie	FMA6 Snares	West Coast 1	West Coast 2
Cook Strait 1		0.0004***	0.0005***	0.0004***	0.0006***	0.0009***	0.0005***	0.0005***	0.0007***	0.0012***	0.0005***	0.0003***
Cook Strait 2	0.0104*		0.0004***	0.0004***	0.0005***	0.0008***	0.0003***	0.0004***	0.0006***	0.0012***	0.0003***	0.0003***
FMA3 A1	0.0000	0.0176***		0.0004***	0.0006***	0.0009***	0.0003***	0.0004***	0.0006***	0.0012***	0.0003***	0.0003***
FMA3 A2	0.0007	0.0000	0.0178*		0.0004***	0.0009***	0.0003***	0.0004***	0.0005***	0.0011***	0.0005***	0.0004***
FMA3 A3	0.0000	0.0041	0.0028	0.0001		0.0009***	0.0004***	0.0004***	0.0004***	0.0009***	0.0006***	0.0005***
FMA3 J1	0.0057	0.0220	0.0063	0.0145	0.0000		0.0009***	0.0008***	0.0011***	0.0016***	0.0008***	0.0008***
FMA J2	0.0090	0.0053	0.0167	0.0067	0.0000	0.0036		0.0003***	0.0005***	0.0011***	0.0004***	0.0003***
FMA4	0.0076*	0.0000	0.0194***	0.0000	0.0003	0.0236	0.0045		0.0005***	0.0012***	0.0003***	0.0003***
FMA6 Norgie	0.0000	0.0000	0.0007	0.0000	0.0000	0.0003	0.0027	0.0056		0.0009***	0.0007***	0.0006***
FMA6 Snares	0.0051	0.0094	0.0080	0.0019	0.0025	0.0099	0.0136*	0.0001	0.0067		0.0012***	0.0010***
West Coast 1	0.0000	0.0057	0.0000	0.0009	0.0000	0.0000	0.0027	0.0032	0.0000	0.0000		0.0003***
West Coast 2	0.0045	0.0045	0.0115*	−0.0002	0.0005	0.0215	0.0074	0.0000	0.0097	0.0000	0.0039	

*p < 0.05, **p < 0.01, ***p < 0.001.

Kmeans clustering, DAPC and LEA analysis results for both the neutral datasets (all hoki and New Zealand only) were very similar to the results of the complete dataset, with two clusters being identified in the dataset containing all sites (a New Zealand cluster and an Australian cluster), and one cluster in the dataset containing only New Zealand sites (Figure 3b,c; Figures S5 and S6). No clustering between juvenile and adult data was present.

#### 3.4.3 Adaptive SNPs for all hoki and New Zealand only

For the dataset containing adaptive SNPs for all hoki, mean nucleotide diversity for New Zealand sites ranged from 0.124 to 0.134, and from 0.419 to 0.442 for Australian sites (Table S2). Mean observed heterozygosity for New Zealand sites ranged from 0.126 to 0.141 and from 0.570 to 0.638 for the two Australian sites. Mean Tajima's *D* ranged from −0.522 to −0.401 for New Zealand sites, and from 1.341 to 1.495 for the two Australian sites. The pairwise *F*_ST_ estimates among New Zealand sites ranged from 0.0000 (*p* = 0.83) to 0.00237 (*p* < 0.001) (mean = 0.00079), and pairwise *F*_ST_ between the two Australian sites was 0.0027 (*p* < 0.001) (Table 5). Pairwise *F*_ST_ distance between all New Zealand and all Australian sites was much higher than for the complete SNP dataset (*F*_ST_ = 0.32393, *p* < 0.001), and when the *F*_ST_ genetic distances were examined as a heatmap, the genetic differentiation between New Zealand and Australian samples remained very clear. In addition, there appeared to be some genetic differentiation occurring within the New Zealand samples, with FMA3 A3, FMA6 Snares and FMA6 Norgie forming their own subcluster within New Zealand; however, as there was poor support for many of these pairwise *F*_ST_ values, this should be interpreted cautiously (Figure S7).

**FIGURE 4:**
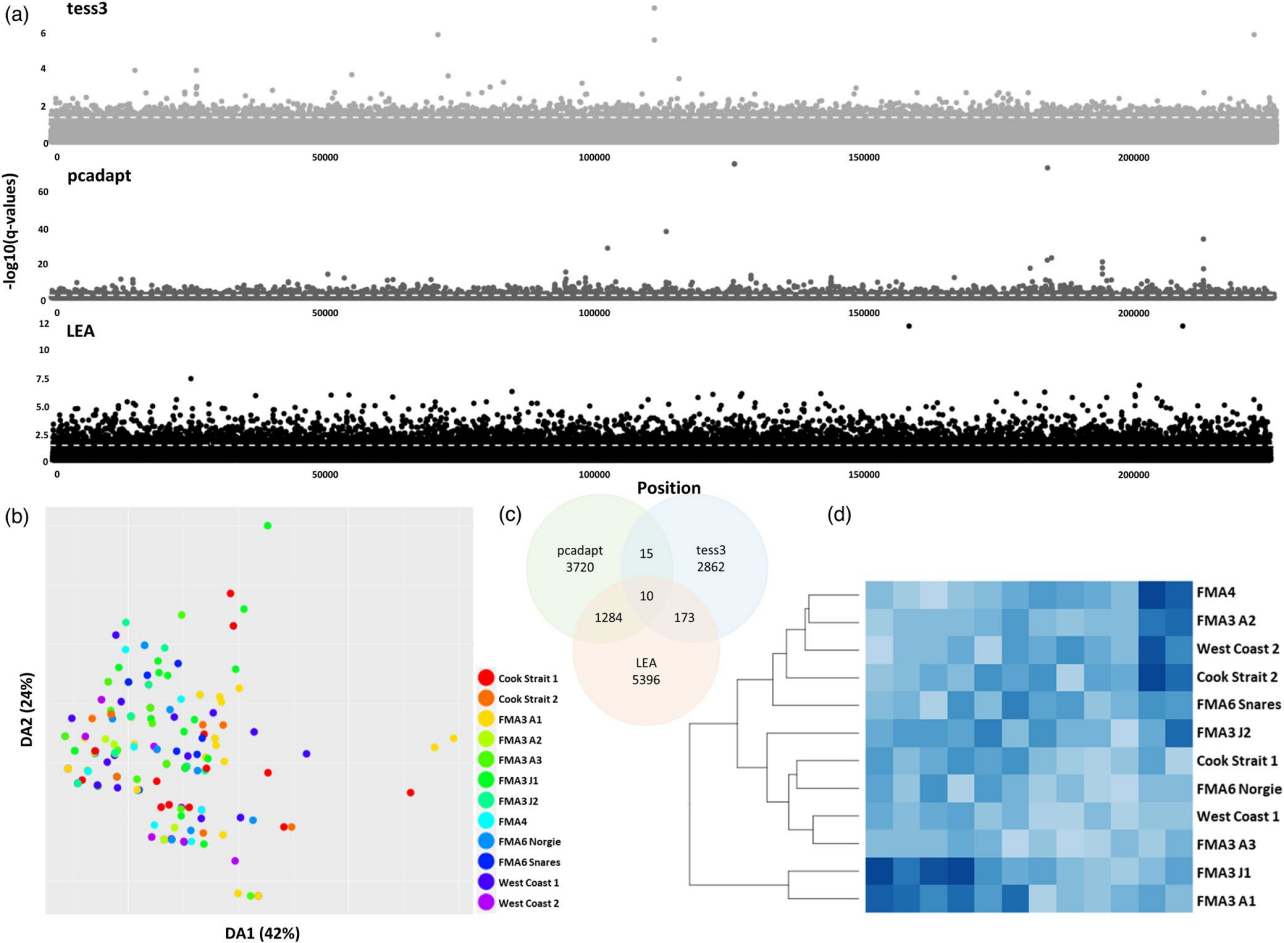
Panel (a) shows the results from Tess3, pcadapt and LEA *q*‐value Manhattan plots of SNPs under selection for the hoki dataset consisting of New Zealand sampling sites only. White dashed lines corresponds to the *Q*‐value threshold of 0.05. (c) the accompanying Venn diagram of putative SNPs under selection for the Tess3, pcadapt and LEA analyses, (b) DAPC scatterplot of DA1 (42%) and DA2 (24%) with points coloured by sampling site for dataset consisting of New Zealand SNPs under selection, (d) pairwise *F*_ST_ heatmap with hierarchical clustering dendrogram–darker blues indicate higher pairwise *F*_ST_ values and lighter blues indicate lower pairwise *F*_ST_ values for dataset consisting of New Zealand SNPs under selection

For the dataset containing adaptive SNPs for only the New Zealand sites, mean nucleotide diversity ranged from 0.092 to 0.192 (Table 4). Mean observed heterozygosity ranged from 0.098 to 0.20, with observed heterozygosity being higher than expected heterozygosity for all sites except FMA3 J2, FMA6 Norgie, FMA3 A3 and Cook Strait 1. Mean Tajima's *D* was negative cross all sites and ranged from −0.653 to −0.028. Pairwise *F*_ST_ distance among New Zealand sites ranged from 0.0000 (*p* = 0.92) to 0.02358 (*p* = 0.16) (mean = 0.00517) (Table 6). When *F*_ST_ distances were plotted as a heatmap, some substructure among populations was visible, with FMA3 A1 and FMA3 J1 forming a cluster and appearing to be genetically separate from the remaining sites; however, again, due to poor support for many of these *F*_ST_ values, this should be interpreted cautiously (Figure 4).

Kmeans clustering, DAPC and LEA analysis results for both the adaptive SNP datasets (all hoki and New Zealand only) were very similar to the results of the complete and neutral datasets, with two clusters being identified in the dataset containing all sites (a New Zealand cluster and an Australian cluster), and one cluster in the dataset containing only New Zealand sites (Figure 4; Figures S8 and S9).

#### 3.4.4 Contemporary and historical *N*_e_ for New Zealand

After thinning the complete SNP dataset of New Zealand samples by 10,000 sites, 2731 SNPs remained for the NeEstimator and SneP analyses. NeEstimator estimated a contemporary LD *N*_e_ of 133,463.4 (95% CI: 38,997; Infinite) (MAF = 0.05). SNeP estimated historical *N*_e_ to be 228,681 999 generations ago (2997–7992 years ago), declining to an *N*_e_ of 42,816 189 generations ago (567–1512) years ago) (Table S3, Figure S10).

## 4 DISCUSSION

Knowledge of the stock structure of important fisheries species is a key component that informs management plans. Marine fish populations typically consist of large numbers of individuals with high dispersal potential, which can result in high gene flow and weak population structure (Bernatchez et al., 2017; Papa et al., 2020). Genomic approaches provide an excellent tool to gain insights into the fine‐scale population structure and can also shed light on the environmental drivers that impose selection on the genome (Bernatchez, 2016). Here we assembled a de novo reference genome and used genome‐wide SNP data to evaluate the stock structure of the deep‐water teleost species hoki (*M. novaezelandiae*). Results showed two clear clusters, one grouping both Australian sites in Tasmania, and another one grouping all New Zealand sites. Within New Zealand, genetic differentiation between sampling locations was weak, indicating reproductive mixing between locations and no clear differentiation between different life history stages (adults vs. juveniles) within New Zealand waters.

The high gene flow and low genetic differentiation between populations characteristic of many teleost species (reviewed in Benestan, 2019; Nielsen et al., 2009) were detected here also for the New Zealand hoki sampling locations. Taking into account both neutral and adaptive SNPs, some evidence for increased genetic differentiation was identified for a small number of subclustering sampling locations based on pairwise *F*_ST_ values. When comparing the dataset including all samples (Australia and New Zealand) with the dataset for only the New Zealand samples, it became evident that the sampling locations within each subcluster were different (FMA3 A3, FMA6 Snares and FMA6 for the whole dataset, and FMA3 A1 and FMA3 J1 for the New Zealand dataset, Figure 4, Figure S4), suggesting the clusters were spurious. Furthermore, all three clustering approaches (DAPC, LEA and Kmeans clustering) using either the neutral or the adaptive dataset did not support *F*_ST_‐based subclusters, and detected only the two clusters differentiating the Australian and New Zealand sampling locations. Together, this evidence indicates that the *F*_ST_ differentiation within New Zealand waters is overall weak and does not carry a strong geographic signal.

While panmixia of New Zealand hoki stocks was inferred, outlier scans including the two Australian sites revealed a large number of loci under putative selection. In total, 2797 SNPs could be identified as outliers that were in common across all three analysis methods. This high number of SNPs under putative selection indicates that the wider Australasian hoki population has a high potential for future genetic adaptation in response to changing environmental conditions. In the context of a rapidly changing world, this high standing adaptive potential may provide this species with a resilience buffer to combat future changes. In contrast to the large number of outlier loci in the overall dataset, when only New Zealand locations were analysed, the number of shared outlier loci between sampling locations fell to only 10 SNPs. This finding could indicate that large‐scale environmental selection selects for different locally adapted genome associations in each cluster; however, further analysis is required to better understand this small number of shared outlier SNPs. Our finding of multiple outlier loci, particularly in the complete dataset, is not in line with previous views on the adaptive ability of marine teleosts (Waples, 1998). In the past, adaptive divergence was widely believed to be rare in marine fishes owing to fewer barriers to gene flow in the marine environment, the often‐long dispersal phase of larvae, and also migratory adults; together, these factors were thought to hinder local adaptation (Kawecki & Ebert, 2004). Our results, and those of other studies in recent years (see for a review Bernatchez, 2016), strongly indicate that this view is no longer warranted and that many marine fishes exhibit signatures of local adaptation. Indeed, recent work suggests that the often‐large population sizes of marine fishes may be fuelling selection, particularly when selection is stable and consistent over large areas, and the selection differentials are large (Hemmer‐Hansen et al., 2007, 2014). This appears to apply strongly to species that are characterized by large populations, meaning that they are generally little affected by random genetic drift, and probably respond to even relatively weak selection, as locally beneficial alleles have a good chance of sweeping through the population.

Our population size estimates of *N*_e_ indicated a general decline in the population size of hoki, from a historical *N*_e_ of 228,681, 999 generations ago, to a *N*_e_ of 42,816, approximately 189 generations ago. These estimates have to be taken with caution, however, as estimation of *N*_e_ values is always plagued by high uncertainty owing to the need to sample a high number of individuals to produce accurate estimates (Blower et al., 2019). In fact, simulations indicate that around 1% of the total number of individuals might have to be sampled to ensure sufficiently precise estimates of *N*_e,_ and our sample size is well below this recommend threshold. Indeed for species with large populations, this would mean that several thousands to millions of individuals would have to be sampled (Marandel et al., 2019). Promising recent developments that rely on the reconstruction of close relatives, such as the close kin mark–recapture (CKMR) approach (Bravington et al., 2016), have demonstrated an improved ability to calculate *N*_e_ with smaller confidence intervals (Ruzzante et al., 2019). However, application of this method to species with large population sizes has been little explored to date and is likely to be cost prohibitive in most cases.

In New Zealand, hoki forms the largest fishery by volume of catch (Papa et al., 2020). Our population genomics findings about hoki stock structure should be interpreted alongside other biological data on the species. Data on spawning patterns, morphometrics and growth rates in part corroborate the currently used two‐stock model of hoki (Hicks et al., 2003; Livingston & Schofield, 1996; Livingston & Sullivan, 2007). However, our findings suggest that the Eastern and Western spawning grounds may undergo sufficient mixing to prevent genetic divergence to the degree where genetic clustering emerges. Furthermore, the finding of overall genetic panmixia and the low rate of pairwise location differentiation together indicate that growth differences could be due to phenotypic plasticity rather than genetically derived stock differences. Despite genetic panmixia in New Zealand, we detected some evidence for selection on some genomic regions, indicating that the overall hoki stock may hold some locally adapted genomic regions that may convey a fitness advantage. In the light of the prediction that changing climatic conditions can negatively affect marine productivity (Lindegren et al., 2018), it will be important for fisheries management to monitor the adaptive potential of this fishery. Future steps may focus on management approaches that seek to maintain locally adaptive variants to avoid depletion of biodiversity that could potentially lead to population decline (Reiss et al., 2009). The demonstration of sustainable management practices is particularly relevant for hoki, because since 2001 it has received Marine Stewardship Council (MSC) Fisheries Certification, which is based on criteria and audit processes that are internationally recognized as the world's highest global scientific standards for Ecosystem Based Fisheries Management (Fisheries, 2019). More detail of temporal and spatial partitioning of the neutral and adaptive variation in this species is needed. Specifically, a genome assembly scaffolded to chromosome scale would enable linkage between variants to be better accounted for, which would facilitate the application of Gene‐Environment Association (GEA) analyses. Nevertheless, the genome assembly presented here is highly contiguous and complete based on the size of the scaffolds obtained, the number of BUSCOs retrieved and the percentage of reads mapping back to the reference. This indicates the SNP set used here is representative of the genome‐wide variation within the species. Additionally, extending the genomic analyses to include structural genomic variation, such as inversions, fusions and copy number variants (CNVs), appears an important next step, as this would allow more holistic capture of the full extent of segregating genomic variation in this species. This will be needed to improve both demography analyses and analyses to identify locally selected variants (Wellenreuther et al., 2019). Knowledge of geographically selected and divergent variants could be further used for the development of a genetic tool applicable to monitoring populations in time and space (Dahle et al., 2018; Hemmer‐Hansen et al., 2019). With the data from this study, it is already possible to use identified divergent SNPs between the New Zealand and Australian clusters to inform hoki seafood traceability, as these could be used to link the catch to its geographic origin (A list of these SNPs is available upon request).

In conclusion, genetic variation at the genome‐wide level is invaluable to identify fish stock structure in fisheries management, and the recent increase in the accessibility and resolution of population genetic data has facilitated the detection of previously unidentified structures, as well as signatures for natural selection in wild populations (Bernatchez, 2016). We highlight the importance of using large numbers of markers distributed across the genome to fully characterize the genetic diversity of marine species. In our current study, this allowed us to separate the Australian and New Zealand clusters, as well as some more subtle differences within New Zealand hoki that otherwise could have been overlooked, and allowed us to scan the genome to detect regions under selection. This study contributes to increasing the genetic knowledge of this important fisheries species, and results can be used to improve our understanding of population dynamics and stock structure. Insights into the species demography is particularly challenging in high gene flow environments, such as many marine fishes, where small genetic differences across most of the genome can mask genetic divergence of strong functional significance. Thus, our study also serves as an example of the increased power offered by population genomics for conservation and management (Allendorf et al., 2010a; Hunter et al., 2018).

## CONFLICT OF INTEREST

The authors declare no competing interests.

## Supporting information

Supplementary Material

## Data Availability

Permission from representatives of the Indigenous Peoples (Māori) was obtained for specimens used in this study. Further studies using this material, raw sequencing data and final genome assembly will require consent from the Māori iwi (tribe) who exercises guardianship for this material according to Aotearoa New Zealand's Treaty of Waitangi and the international Nagoya protocol on the rights of indigenous peoples. Raw and analysed data are available through the Genomics Aotearoa data repository at https://www.genomics‐aotearoa.org.nz/data. Access to these data will require permission from Te Ohu Kaimoana ( ika@teohu.maori.nz ).
