# Supplementary material for: Genome‐wide analysis reveals the genetic stock structure of hoki (Macruronus novaezelandiae)
Source: Evol Appl. 2021 Nov 23;14(12):2848–63. doi: 10.1111/eva.13317 (PMC8674887; doi:10.1111/eva.13317)

# Supplementary Material: Genome-wide analysis reveals the genetic stock structure of hoki (*Macruronus novaezelandiae)*

Emily Koot^1^, Chen Wu^2^, Igor Ruza^3^, Elena Hilario^2^, Roy Storey^4^, Richard Wells^5^, David Chagné^1^, and Maren Wellenreuther^3,6,*^

**Table S1: A summary of the sequencing data for the 510 sampled hoki.**

| Sample | Number of reads | Total bases | Mapping rate to reference genome |
| --- | --- | --- | --- |
| BCP4874 | 119827880 | 17974182000 | 92.82% |
| BCP4875 | 62357666 | 9353649900 | 92.97% |
| BCP4876 | 106220147 | 15933022050 | 93.01% |
| BCP4877 | 83587021 | 12538053150 | 93.08% |
| BCP4878 | 75682786 | 11352417900 | 92.76% |
| BCP4879 | 91249166 | 13687374900 | 92.87% |
| BCP4880 | 69562625 | 10434393750 | 92.75% |
| BCP4881 | 64763240 | 9714486000 | 92.14% |
| BCP4882 | 63700873 | 9555130950 | 92.72% |
| BCP4883 | 64539270 | 9680890500 | 92.61% |
| BCP4884 | 68588101 | 10288215150 | 93.32% |
| BCP4885 | 85699369 | 12854905350 | 92.53% |
| BCP4886 | 65134490 | 9770173500 | 92.61% |
| BCP4887 | 78144802 | 11721720300 | 92.63% |
| BCP4888 | 113787464 | 17068119600 | 92.58% |
| BCP4889 | 95284651 | 14292697650 | 92.71% |
| BCP4890 | 71885774 | 10782866100 | 93.74% |
| BCP4891 | 62680553 | 9402082950 | 92.57% |
| BCP4892 | 79090157 | 11863523550 | 0.00% |
| BCP4893 | 76438050 | 11465707500 | 92.57% |
| BCP4894 | 61940770 | 9291115500 | 92.67% |
| BCP4895 | 67602351 | 10140352650 | 92.53% |
| BCP4896 | 67331479 | 10099721850 | 92.66% |
| BCP4897 | 99311905 | 14896785750 | 93.35% |
| BCP4898 | 136688517 | 20503277550 | 92.97% |
| BCP4899 | 78789396 | 11818409400 | 92.78% |
| BCP4900 | 33995569 | 5099335350 | 92.10% |
| BCP4901 | 100012415 | 15001862250 | 92.84% |
| BCP4902 | 65641428 | 9846214200 | 92.69% |
| BCP4903 | 77545804 | 11631870600 | 92.64% |
| BCP4904 | 79905071 | 11985760650 | 92.65% |
| BCP4905 | 72390509 | 10858576350 | 92.47% |
| BCP4906 | 95791511 | 14368726650 | 94.69% |
| BCP4907 | 69527283 | 10429092450 | 92.30% |
| BCP4908 | 84805200 | 12720780000 | 92.22% |
| BCP4909 | 71898323 | 10784748450 | 92.35% |
| BCP4910 | 85179018 | 12776852700 | 92.97% |
| BCP4911 | 70146353 | 10521952950 | 92.40% |
| BCP4912 | 86104445 | 12915666750 | 92.62% |
| BCP4913 | 92556492 | 13883473800 | 92.09% |
| BCP5174 | 82302686 | 12345402900 | 91.97% |
| BCP5175 | 90728366 | 13609254900 | 92.61% |
| BCP5176 | 76286641 | 11442996150 | 92.90% |
| BCP5177 | 66578339 | 9986750850 | 92.44% |
| BCP5178 | 92224707 | 13833706050 | 93.45% |
| BCP5179 | 47929968 | 7189495200 | 92.47% |
| BCP5180 | 70893543 | 10634031450 | 92.37% |
| BCP5181 | 83512939 | 12526940850 | 93.64% |
| BCP5182 | 67135992 | 10070398800 | 93.03% |
| BCP5183 | 125381290 | 18807193500 | 92.42% |
| BCP5184 | 84314055 | 12647108250 | 93.13% |
| BCP5185 | 71889076 | 10783361400 | 93.18% |
| BCP5186 | 63350614 | 9502592100 | 92.37% |
| BCP5187 | 63677110 | 9551566500 | 92.99% |
| BCP5188 | 103086455 | 15462968250 | 93.74% |
| BCP5189 | 65704765 | 9855714750 | 92.64% |
| BCP5190 | 67114261 | 10067139150 | 92.59% |
| BCP5191 | 105387344 | 15808101600 | 94.53% |
| BCP5192 | 65912705 | 9886905750 | 93.15% |
| BCP5193 | 77382312 | 11607346800 | 92.75% |
| BCP5194 | 75476223 | 11321433450 | 92.52% |
| BCP5195 | 84727366 | 12709104900 | 92.57% |
| BCP5196 | 79733890 | 11960083500 | 93.47% |
| BCP5197 | 79337518 | 11900627700 | 93.52% |
| BCP5198 | 69864221 | 10479633150 | 92.50% |
| BCP5199 | 121029054 | 18154358100 | 93.02% |
| BCP5200 | 96671394 | 14500709100 | 93.21% |
| BCP5201 | 74168019 | 11125202850 | 92.59% |
| BCP5202 | 68270100 | 10240515000 | 92.38% |
| BCP5203 | 69809584 | 10471437600 | 92.46% |
| BCP5204 | 62537945 | 9380691750 | 92.64% |
| BCP5205 | 62880675 | 9432101250 | 92.56% |
| BCP5206 | 88492847 | 13273927050 | 93.23% |
| BCP5207 | 67103224 | 10065483600 | 92.82% |
| BCP5208 | 72972741 | 10945911150 | 92.46% |
| BCP5209 | 67985162 | 10197774300 | 92.58% |
| BCP5210 | 87152793 | 13072918950 | 93.42% |
| BCP5211 | 75062997 | 11259449550 | 92.56% |
| BCP5212 | 75327047 | 11299057050 | 92.66% |
| BCP5213 | 121412351 | 18211852650 | 92.42% |
| BCP5474 | 73506303 | 11025945450 | 92.31% |
| BCP5475 | 75815050 | 11372257500 | 92.68% |
| BCP5476 | 105814679 | 15872201850 | 92.11% |
| BCP5477 | 81170542 | 12175581300 | 92.52% |
| BCP5478 | 103148938 | 15472340700 | 93.50% |
| BCP5479 | 83400286 | 12510042900 | 93.51% |
| BCP5480 | 90837500 | 13625625000 | 92.40% |
| BCP5481 | 73125470 | 10968820500 | 92.27% |
| BCP5482 | 76669057 | 11500358550 | 92.74% |
| BCP5483 | 66871594 | 10030739100 | 92.05% |
| BCP5484 | 63144784 | 9471717600 | 91.77% |
| BCP5485 | 81977159 | 12296573850 | 92.96% |
| BCP5486 | 63391121 | 9508668150 | 92.23% |
| BCP5487 | 60957016 | 9143552400 | 92.16% |
| BCP5488 | 81063815 | 12159572250 | 92.26% |
| BCP5489 | 105663701 | 15849555150 | 92.47% |
| BCP5490 | 80781822 | 12117273300 | 92.42% |
| BCP5491 | 72463261 | 10869489150 | 92.54% |
| BCP5492 | 63377867 | 9506680050 | 92.55% |
| BCP5493 | 61804221 | 9270633150 | 92.92% |
| BCP5494 | 71232471 | 10684870650 | 92.98% |
| BCP5495 | 84986772 | 12748015800 | 93.48% |
| BCP5496 | 81470437 | 12220565550 | 92.52% |
| BCP5497 | 79517360 | 11927604000 | 92.78% |
| BCP5498 | 108719098 | 16307864700 | 92.54% |
| BCP5499 | 101217166 | 15182574900 | 93.04% |
| BCP5500 | 94444829 | 14166724350 | 93.06% |
| BCP5501 | 79369539 | 11905430850 | 92.91% |
| BCP5502 | 97018070 | 14552710500 | 92.90% |
| BCP5503 | 66469434 | 9970415100 | 92.37% |
| BCP5504 | 68989766 | 10348464900 | 92.45% |
| BCP5505 | 88712051 | 13306807650 | 92.67% |
| BCP5506 | 79403973 | 11910595950 | 92.41% |
| BCP5507 | 84518299 | 12677744850 | 92.63% |
| BCP5508 | 61028671 | 9154300650 | 91.60% |
| BCP5509 | 68723934 | 10308590100 | 91.11% |
| BCP5510 | 83151930 | 12472789500 | 92.21% |
| BCP5511 | 91963444 | 13794516600 | 92.83% |
| BCP5512 | 74808167 | 11221225050 | 91.99% |
| BCP5513 | 103496009 | 15524401350 | 93.81% |
| BCP5574 | 64267329 | 9640099350 | 92.82% |
| BCP5575 | 77568694 | 11635304100 | 93.17% |
| BCP5576 | 87564318 | 13134647700 | 93.47% |
| BCP5577 | 73858786 | 11078817900 | 92.38% |
| BCP5578 | 70491957 | 10573793550 | 93.89% |
| BCP5579 | 67939990 | 10190998500 | 92.41% |
| BCP5580 | 65401310 | 9810196500 | 92.04% |
| BCP5581 | 62993080 | 9448962000 | 92.34% |
| BCP5582 | 103906074 | 15585911100 | 92.93% |
| BCP5583 | 88172345 | 13225851750 | 92.62% |
| BCP5584 | 77566471 | 11634970650 | 92.47% |
| BCP5585 | 88931357 | 13339703550 | 92.27% |
| BCP5586 | 60983569 | 9147535350 | 92.22% |
| BCP5587 | 68328687 | 10249303050 | 92.80% |
| BCP5588 | 101501694 | 15225254100 | 93.57% |
| BCP5589 | 97882327 | 14682349050 | 93.58% |
| BCP5590 | 114077316 | 17111597400 | 92.70% |
| BCP5591 | 70324424 | 10548663600 | 93.01% |
| BCP5592 | 72045036 | 10806755400 | 92.96% |
| BCP5593 | 81535477 | 12230321550 | 92.53% |
| BCP5594 | 71631820 | 10744773000 | 92.63% |
| BCP5595 | 89928397 | 13489259550 | 92.53% |
| BCP5596 | 70109998 | 10516499700 | 92.89% |
| BCP5597 | 74275879 | 11141381850 | 92.75% |
| BCP5598 | 85412102 | 12811815300 | 93.16% |
| BCP5599 | 61813015 | 9271952250 | 92.92% |
| BCP5600 | 61932657 | 9289898550 | 92.92% |
| BCP5601 | 79860391 | 11979058650 | 92.48% |
| BCP5602 | 76120242 | 11418036300 | 92.71% |
| BCP5603 | 81073755 | 12161063250 | 92.87% |
| BCP5604 | 79019001 | 11852850150 | 92.66% |
| BCP5605 | 66876686 | 10031502900 | 92.88% |
| BCP5606 | 67503715 | 10125557250 | 92.68% |
| BCP5607 | 84850614 | 12727592100 | 92.59% |
| BCP5608 | 84398999 | 12659849850 | 92.71% |
| BCP5609 | 72044427 | 10806664050 | 92.84% |
| BCP5610 | 72118645 | 10817796750 | 93.02% |
| BCP5611 | 66206074 | 9930911100 | 93.57% |
| BCP5612 | 94293962 | 14144094300 | 93.27% |
| BCP5613 | 63106328 | 9465949200 | 93.13% |
| BCP5674 | 74183962 | 11127594300 | 92.53% |
| BCP5675 | 66925652 | 10038847800 | 92.46% |
| BCP5676 | 71706476 | 10755971400 | 93.55% |
| BCP5677 | 78323957 | 11748593550 | 93.61% |
| BCP5678 | 65484492 | 9822673800 | 93.15% |
| BCP5679 | 82904217 | 12435632550 | 92.64% |
| BCP5680 | 82747713 | 12412156950 | 92.39% |
| BCP5681 | 91907940 | 13786191000 | 94.91% |
| BCP5682 | 102406000 | 15360900000 | 92.61% |
| BCP5683 | 71687384 | 10753107600 | 91.78% |
| BCP5684 | 94039520 | 14105928000 | 92.59% |
| BCP5685 | 63797531 | 9569629650 | 92.64% |
| BCP5686 | 74176107 | 11126416050 | 92.51% |
| BCP5687 | 73526142 | 11028921300 | 92.58% |
| BCP5688 | 74855878 | 11228381700 | 92.45% |
| BCP5689 | 73347547 | 11002132050 | 92.59% |
| BCP5690 | 74004215 | 11100632250 | 92.44% |
| BCP5691 | 81880788 | 12282118200 | 92.88% |
| BCP5692 | 84962856 | 12744428400 | 93.21% |
| BCP5693 | 74785881 | 11217882150 | 92.57% |
| BCP5694 | 71278236 | 10691735400 | 92.48% |
| BCP5695 | 88833692 | 13325053800 | 93.13% |
| BCP5696 | 81428466 | 12214269900 | 93.05% |
| BCP5697 | 77531848 | 11629777200 | 92.48% |
| BCP5698 | 73460313 | 11019046950 | 92.92% |
| BCP5699 | 63252441 | 9487866150 | 93.01% |
| BCP5700 | 77145290 | 11571793500 | 92.05% |
| BCP5701 | 76783568 | 11517535200 | 92.58% |
| BCP5702 | 129685720 | 19452858000 | 94.24% |
| BCP5703 | 57953691 | 8693053650 | 92.62% |
| BCP5704 | 70470444 | 10570566600 | 92.84% |
| BCP5705 | 82836930 | 12425539500 | 93.38% |
| BCP5706 | 72009679 | 10801451850 | 93.08% |
| BCP5707 | 79232455 | 11884868250 | 92.85% |
| BCP5708 | 67243116 | 10086467400 | 92.59% |
| BCP5709 | 76945522 | 11541828300 | 92.73% |
| BCP5710 | 68201376 | 10230206400 | 92.44% |
| BCP5711 | 77728125 | 11659218750 | 92.88% |
| BCP5712 | 74493595 | 11174039250 | 92.55% |
| BCP5713 | 109482987 | 16422448050 | 94.43% |
| BCP5774 | 87029186 | 13054377900 | 94.55% |
| BCP5775 | 93236709 | 13985506350 | 92.71% |
| BCP5776 | 76173523 | 11426028450 | 92.37% |
| BCP5777 | 105750478 | 15862571700 | 92.84% |
| BCP5778 | 124185118 | 18627767700 | 92.58% |
| BCP5779 | 79819339 | 11972900850 | 93.70% |
| BCP5780 | 90327245 | 13549086750 | 93.19% |
| BCP5781 | 94189701 | 14128455150 | 94.43% |
| BCP5782 | 66752479 | 10012871850 | 93.55% |
| BCP5783 | 61629484 | 9244422600 | 92.28% |
| BCP5784 | 69683251 | 10452487650 | 92.36% |
| BCP5785 | 111295009 | 16694251350 | 93.73% |
| BCP5786 | 89756922 | 13463538300 | 93.28% |
| BCP5787 | 63872707 | 9580906050 | 92.93% |
| BCP5788 | 101806592 | 15270988800 | 94.18% |
| BCP5789 | 85340886 | 12801132900 | 92.48% |
| BCP5790 | 71865515 | 10779827250 | 92.37% |
| BCP5791 | 85938941 | 12890841150 | 92.87% |
| BCP5792 | 63115187 | 9467278050 | 92.36% |
| BCP5793 | 88526654 | 13278998100 | 94.14% |
| BCP5794 | 75627278 | 11344091700 | 92.99% |
| BCP5795 | 85066762 | 12760014300 | 92.74% |
| BCP5796 | 65314846 | 9797226900 | 92.37% |
| BCP5797 | 99018411 | 14852761650 | 94.07% |
| BCP5798 | 72918677 | 10937801550 | 92.28% |
| BCP5799 | 69371174 | 10405676100 | 92.08% |
| BCP5800 | 94685343 | 14202801450 | 93.90% |
| BCP5802 | 79338301 | 11900745150 | 93.02% |
| BCP5803 | 79929028 | 11989354200 | 92.37% |
| BCP5804 | 67449285 | 10117392750 | 92.44% |
| BCP5805 | 95012719 | 14251907850 | 92.89% |
| BCP5806 | 61141198 | 9171179700 | 92.26% |
| BCP5807 | 77964658 | 11694698700 | 92.48% |
| BCP5808 | 63985946 | 9597891900 | 92.44% |
| BCP5809 | 79931411 | 11989711650 | 93.39% |
| BCP5810 | 103957458 | 15593618700 | 92.77% |
| BCP5811 | 64968155 | 9745223250 | 92.94% |
| BCP5812 | 64345589 | 9651838350 | 92.42% |
| BCP5813 | 78560132 | 11784019800 | 93.02% |
| BCP5814 | 71664497 | 10749674550 | 93.29% |
| BCP5874 | 68829788 | 10324468200 | 92.53% |
| BCP5875 | 70114448 | 10517167200 | 92.75% |
| BCP5876 | 54316659 | 8147498850 | 92.13% |
| BCP5877 | 69012749 | 10351912350 | 92.41% |
| BCP5878 | 85185259 | 12777788850 | 92.28% |
| BCP5879 | 63749565 | 9562434750 | 92.88% |
| BCP5880 | 63379522 | 9506928300 | 93.40% |
| BCP5881 | 83878989 | 12581848350 | 92.48% |
| BCP5882 | 76345500 | 11451825000 | 93.98% |
| BCP5883 | 70655684 | 10598352600 | 92.47% |
| BCP5884 | 97976578 | 14696486700 | 94.40% |
| BCP5885 | 74049553 | 11107432950 | 91.99% |
| BCP5886 | 72332260 | 10849839000 | 92.39% |
| BCP5887 | 79425116 | 11913767400 | 93.01% |
| BCP5888 | 82454996 | 12368249400 | 92.34% |
| BCP5889 | 94311110 | 14146666500 | 93.68% |
| BCP5890 | 87657689 | 13148653350 | 92.79% |
| BCP5891 | 73966021 | 11094903150 | 92.48% |
| BCP5892 | 73071085 | 10960662750 | 92.69% |
| BCP5893 | 65930844 | 9889626600 | 92.80% |
| BCP5894 | 80441219 | 12066182850 | 92.28% |
| BCP5895 | 65045247 | 9756787050 | 91.93% |
| BCP5896 | 72343216 | 10851482400 | 92.61% |
| BCP5897 | 86649283 | 12997392450 | 93.07% |
| BCP5898 | 79211216 | 11881682400 | 92.46% |
| BCP5899 | 62816096 | 9422414400 | 92.25% |
| BCP5900 | 98102147 | 14715322050 | 94.46% |
| BCP5901 | 72214892 | 10832233800 | 92.01% |
| BCP5902 | 80682608 | 12102391200 | 93.28% |
| BCP5903 | 78445169 | 11766775350 | 92.33% |
| BCP5904 | 75620700 | 11343105000 | 92.26% |
| BCP5905 | 70801164 | 10620174600 | 92.47% |
| BCP5906 | 80656057 | 12098408550 | 94.56% |
| BCP5907 | 64733113 | 9709966950 | 92.27% |
| BCP5908 | 61959140 | 9293871000 | 92.42% |
| BCP5909 | 73904792 | 11085718800 | 92.39% |
| BCP5910 | 71616150 | 10742422500 | 92.42% |
| BCP5911 | 65461907 | 9819286050 | 92.22% |
| BCP5912 | 65932857 | 9889928550 | 90.60% |
| BCP5913 | 82077821 | 12311673150 | 92.64% |
| BCP5974 | 69687141 | 10453071150 | 91.58% |
| BCP5975 | 85139182 | 12770877300 | 92.50% |
| BCP5976 | 80737509 | 12110626350 | 92.37% |
| BCP5977 | 77127139 | 11569070850 | 92.58% |
| BCP5978 | 101062659 | 15159398850 | 94.38% |
| BCP5979 | 81126128 | 12168919200 | 92.81% |
| BCP5980 | 68199185 | 10229877750 | 92.56% |
| BCP5981 | 70264017 | 10539602550 | 93.84% |
| BCP5982 | 85528706 | 12829305900 | 92.61% |
| BCP5983 | 79071503 | 11860725450 | 93.79% |
| BCP5984 | 83188599 | 12478289850 | 92.56% |
| BCP5985 | 81912347 | 12286852050 | 92.60% |
| BCP5986 | 88370612 | 13255591800 | 92.83% |
| BCP5987 | 64533560 | 9680034000 | 91.14% |
| BCP5988 | 86621307 | 12993196050 | 93.19% |
| BCP5989 | 64240652 | 9636097800 | 92.78% |
| BCP5990 | 81023433 | 12153514950 | 92.42% |
| BCP5991 | 72366515 | 10854977250 | 92.93% |
| BCP5992 | 68779142 | 10316871300 | 92.99% |
| BCP5993 | 62843972 | 9426595800 | 92.22% |
| BCP5994 | 84451742 | 12667761300 | 93.21% |
| BCP5995 | 81293449 | 12194017350 | 92.66% |
| BCP5996 | 75823592 | 11373538800 | 92.10% |
| BCP5997 | 79840806 | 11976120900 | 92.40% |
| BCP5998 | 78925226 | 11838783900 | 92.54% |
| BCP5999 | 83916750 | 12587512500 | 92.46% |
| BCP6000 | 78731332 | 11809699800 | 92.64% |
| BCP6001 | 82636806 | 12395520900 | 93.04% |
| BCP6002 | 61872832 | 9280924800 | 92.33% |
| BCP6003 | 76675630 | 11501344500 | 92.62% |
| BCP6004 | 96617303 | 14492595450 | 94.28% |
| BCP6005 | 80624793 | 12093718950 | 93.60% |
| BCP6006 | 73804950 | 11070742500 | 92.58% |
| BCP6007 | 94336593 | 14150488950 | 93.73% |
| BCP6008 | 75576407 | 11336461050 | 92.70% |
| BCP6009 | 88412760 | 13261914000 | 93.23% |
| BCP6010 | 77702601 | 11655390150 | 93.03% |
| BCP6011 | 92585340 | 13887801000 | 92.64% |
| BCP6012 | 140403157 | 21060473550 | 92.17% |
| BCP6013 | 68915751 | 10337362650 | 92.39% |
| FISH00W4 | 94377774 | 14156666100 | 93.68% |
| FISH00W5 | 84790072 | 12718510800 | 93.43% |
| FISH00W6 | 88259602 | 13238940300 | 92.91% |
| FISH00W7 | 78307278 | 11746091700 | 93.10% |
| FISH00W8 | 80173905 | 12026085750 | 89.95% |
| FISH00W9 | 79271210 | 11890681500 | 93.41% |
| FISH00WA | 84233159 | 12634973850 | 93.06% |
| FISH00WB | 66396087 | 9959413050 | 91.88% |
| FISH00WC | 72693912 | 10904086800 | 92.36% |
| FISH00WD | 75567656 | 11335148400 | 91.69% |
| FISH00WE | 86086427 | 12912964050 | 91.81% |
| FISH00WF | 62184722 | 9327708300 | 91.50% |
| FISH00WG | 80146688 | 12022003200 | 91.66% |
| FISH00WH | 68767841 | 10315176150 | 93.34% |
| FISH00WJ | 62810813 | 9421621950 | 93.25% |
| FISH00WK | 89874196 | 13481129400 | 93.69% |
| FISH00WL | 68367030 | 10255054500 | 92.76% |
| FISH00WM | 69947086 | 10492062900 | 92.72% |
| FISH00WN | 83556297 | 12533444550 | 94.72% |
| FISH00WP | 82887632 | 12433144800 | 93.14% |
| FISH00WR | 78323376 | 11748506400 | 92.42% |
| FISH00WS | 112469838 | 16870475700 | 92.38% |
| FISH00WT | 63900285 | 9585042750 | 93.01% |
| FISH00WV | 97355289 | 14603293350 | 92.34% |
| FISH00WW | 70507776 | 10576166400 | 92.21% |
| FISH00WX | 90619969 | 13592995350 | 94.07% |
| FISH00WY | 77913697 | 11687054550 | 92.01% |
| FISH00WZ | 79871425 | 11980713750 | 93.00% |
| FISH00X0 | 72858920 | 10928838000 | 93.67% |
| FISH00X1 | 85713521 | 12857028150 | 93.31% |
| FISH012C | 81411591 | 12211738650 | 89.76% |
| FISH012D | 80298405 | 12044760750 | 92.52% |
| FISH012E | 73733999 | 11060099850 | 92.58% |
| FISH012F | 97780704 | 14667105600 | 92.37% |
| FISH012G | 86789383 | 13018407450 | 91.66% |
| FISH012H | 66147571 | 9922135650 | 92.01% |
| FISH012J | 71121575 | 10668236250 | 92.27% |
| FISH012K | 92341069 | 13851160350 | 91.85% |
| FISH012L | 69693382 | 10454007300 | 91.95% |
| FISH012M | 117053675 | 17558051250 | 91.53% |
| FISH012N | 79486020 | 11922903000 | 92.90% |
| FISH012P | 85512696 | 12826904400 | 93.03% |
| FISH012R | 93319607 | 13997941050 | 93.37% |
| FISH012S | 103804749 | 15570712350 | 92.52% |
| FISH012T | 71426604 | 10713990600 | 91.61% |
| FISH012V | 106592090 | 15988813500 | 93.97% |
| FISH012W | 86807021 | 13021053150 | 93.16% |
| FISH012X | 99258854 | 14888828100 | 93.38% |
| FISH012Y | 91320162 | 13698024300 | 92.37% |
| FISH012Z | 76931405 | 11539710750 | 90.31% |
| FISH0130 | 61652837 | 9247925550 | 92.83% |
| FISH0131 | 67993843 | 10199076450 | 93.38% |
| FISH0132 | 93103860 | 13965579000 | 92.44% |
| FISH0133 | 113170919 | 16975637850 | 94.32% |
| FISH0134 | 74593475 | 11189021250 | 93.24% |
| FISH0135 | 89521154 | 13428173100 | 93.18% |
| FISH0136 | 93010774 | 13951616100 | 94.13% |
| FISH0137 | 87448389 | 13117258350 | 93.23% |
| FISH0138 | 109682190 | 16452328500 | 93.98% |
| FISH0139 | 97798925 | 14669838750 | 92.67% |
| FISH01VD | 70317498 | 10547624700 | 92.87% |
| FISH01VE | 84405392 | 12660808800 | 93.71% |
| FISH01VF | 68391325 | 10258698750 | 91.92% |
| FISH01VG | 92264293 | 13839643950 | 92.29% |
| FISH01VH | 74840427 | 11226064050 | 91.66% |
| FISH01VJ | 83667240 | 12550086000 | 92.35% |
| FISH01VK | 92051365 | 13807704750 | 93.54% |
| FISH01VL | 75467444 | 11320116600 | 93.34% |
| FISH01VM | 73018029 | 10952704350 | 92.36% |
| FISH01VN | 63056871 | 9458530650 | 92.40% |
| FISH01VP | 103589852 | 15538477800 | 93.73% |
| FISH01VR | 123503455 | 18525518250 | 93.21% |
| FISH01VS | 87137733 | 13070659950 | 92.63% |
| FISH01VT | 74797271 | 11219590650 | 93.08% |
| FISH01VV | 65480026 | 9822003900 | 92.34% |
| FISH01VW | 135327638 | 20299145700 | 94.17% |
| FISH01VX | 80265335 | 12039800250 | 91.93% |
| FISH01VY | 89763314 | 13464497100 | 93.26% |
| FISH01VZ | 122395947 | 18359392050 | 94.20% |
| FISH01W0 | 91244271 | 13686640650 | 93.44% |
| FISH01W1 | 62256398 | 9338459700 | 92.50% |
| FISH01W2 | 103993355 | 15599003250 | 93.81% |
| FISH01W3 | 102467779 | 15370166850 | 93.74% |
| FISH01W4 | 76690949 | 11503642350 | 93.42% |
| FISH01W5 | 77148064 | 11572209600 | 92.57% |
| FISH01W6 | 68074385 | 10211157750 | 92.69% |
| FISH01W7 | 120687952 | 18103192800 | 91.54% |
| FISH01W8 | 70035386 | 10505307900 | 92.26% |
| FISH01W9 | 104008217 | 15601232550 | 93.86% |
| FISH01WA | 74124684 | 11118702600 | 92.09% |
| FISH024V | 72995759 | 10949363850 | 92.69% |
| FISH0255 | 64495606 | 9674340900 | 92.58% |
| FISH0259 | 102993870 | 15449080500 | 92.54% |
| FISH025B | 98839175 | 14825876250 | 92.68% |
| FISH025D | 75833850 | 11375077500 | 92.55% |
| FISH025E | 78835294 | 11825294100 | 92.53% |
| FISH025G | 72331421 | 10849713150 | 92.70% |
| FISH025L | 63076217 | 9461432550 | 92.76% |
| FISH025M | 65023494 | 9753524100 | 92.77% |
| FISH025P | 75857206 | 11378580900 | 92.64% |
| FISH025R | 77803527 | 11670529050 | 92.73% |
| FISH025V | 74943331 | 11241499650 | 92.69% |
| FISH025W | 86361961 | 12954294150 | 92.20% |
| FISH025Y | 86201445 | 12930216750 | 92.81% |
| FISH0260 | 69104110 | 10365616500 | 92.58% |
| FISH0261 | 77904938 | 11685740700 | 92.67% |
| FISH0262 | 70909082 | 10636362300 | 92.71% |
| FISH0263 | 62442452 | 9366367800 | 92.79% |
| FISH0267 | 65568016 | 9835202400 | 92.81% |
| FISH0268 | 70128844 | 10519326600 | 92.55% |
| FISH0269 | 73468266 | 11020239900 | 92.57% |
| FISH026A | 96235212 | 14435281800 | 92.61% |
| FISH026B | 89875180 | 13481277000 | 92.42% |
| FISH026D | 108921160 | 16338174000 | 92.46% |
| FISH026E | 79867379 | 11980106850 | 92.72% |
| FISH026F | 79119554 | 11867933100 | 92.78% |
| FISH026G | 72691231 | 10903684650 | 92.63% |
| FISH026H | 63150880 | 9472632000 | 92.41% |
| FISH026M | 80214811 | 12032221650 | 92.44% |
| FISH026N | 65835939 | 9875390850 | 92.67% |
| FISH026P | 74278635 | 11141795250 | 92.21% |
| FISH026W | 74082020 | 11112303000 | 93.89% |
| FISH026Y | 63011149 | 9451672350 | 92.68% |
| FISH0271 | 65441488 | 9816223200 | 92.56% |
| FISH0272 | 71206772 | 10681015800 | 92.56% |
| FISH0276 | 70031710 | 10504756500 | 92.86% |
| FISH0278 | 81608949 | 12241342350 | 92.58% |
| FISH027A | 91223621 | 13683543150 | 92.64% |
| FISH027B | 76607999 | 11491199850 | 92.44% |
| FISH027D | 87417419 | 13112612850 | 92.40% |
| FISH027Y | 73610676 | 11041601400 | 91.59% |
| FISH027Z | 63762058 | 9564308700 | 92.11% |
| FISH0280 | 74212879 | 11131931850 | 92.11% |
| FISH0281 | 84631289 | 12694693350 | 92.07% |
| FISH0282 | 92715834 | 13907375100 | 92.04% |
| FISH0283 | 75734353 | 11360152950 | 92.58% |
| FISH0284 | 78439518 | 11765927700 | 92.17% |
| FISH0285 | 75563528 | 11334529200 | 91.12% |
| FISH0286 | 105889511 | 15883426650 | 92.24% |
| FISH0287 | 75966403 | 11394960450 | 91.89% |
| FISH0288 | 80351533 | 12052729950 | 92.80% |
| FISH0289 | 116239938 | 17435990700 | 81.49% |
| FISH028A | 85974890 | 12896233500 | 92.31% |
| FISH028B | 98221290 | 14733193500 | 91.98% |
| FISH028C | 117887233 | 17683084950 | 89.82% |
| FISH028D | 96267471 | 14440120650 | 91.05% |
| FISH028E | 85525119 | 12828767850 | 93.00% |
| FISH028F | 63995891 | 9599383650 | 91.50% |
| FISH028G | 87016141 | 13052421150 | 92.81% |
| FISH028H | 115494813 | 17324221950 | 92.96% |
| FISH028J | 83672339 | 12550850850 | 93.48% |
| FISH028K | 84773056 | 12715958400 | 92.48% |
| FISH028L | 102511266 | 15376689900 | 93.08% |
| FISH028M | 90664094 | 13599614100 | 90.18% |
| FISH028N | 80628155 | 12094223250 | 92.62% |
| FISH028P | 78806236 | 11820935400 | 92.59% |
| FISH028R | 84861342 | 12729201300 | 91.44% |
| FISH028S | 71647815 | 10747172250 | 92.78% |
| FISH028T | 101771534 | 15265730100 | 93.30% |
| FISH028V | 97070901 | 14560635150 | 93.23% |
| FISH02B2 | 94986698 | 14248004700 | 85.36% |
| FISH02B3 | 88689196 | 13303379400 | 92.76% |
| FISH02B4 | 69287959 | 10393193850 | 91.11% |
| FISH02B5 | 76229376 | 11434406400 | 93.16% |
| FISH02B6 | 62951053 | 9442657950 | 92.14% |
| FISH02B7 | 76204872 | 11430730800 | 92.10% |
| FISH02B8 | 66199791 | 9929968650 | 91.99% |
| FISH02B9 | 66172988 | 9925948200 | 90.76% |
| FISH02BA | 77822876 | 11673431400 | 92.37% |
| FISH02BB | 66310411 | 9946561650 | 91.98% |
| FISH02BC | 67942303 | 10191345450 | 91.35% |
| FISH02BD | 98589835 | 14788475250 | 92.90% |
| FISH02BE | 71804915 | 10770737250 | 90.81% |
| FISH02BF | 108786167 | 16317925050 | 92.24% |
| FISH02BG | 61740536 | 9261080400 | 89.03% |
| FISH02BH | 64183889 | 9627583350 | 91.65% |
| FISH02BJ | 82943205 | 12441480750 | 92.82% |
| FISH02BK | 73767735 | 11065160250 | 91.85% |
| FISH02BL | 87436283 | 13115442450 | 92.70% |
| FISH02BM | 71029978 | 10654496700 | 92.30% |
| FISH02BN | 90663169 | 13599475350 | 90.13% |
| FISH02BP | 77918795 | 11687819250 | 91.89% |
| FISH02BR | 88745487 | 13311823050 | 92.35% |
| FISH02BS | 76728302 | 11509245300 | 92.18% |
| FISH02BT | 93215872 | 13982380800 | 89.38% |
| FISH02BV | 92261487 | 13839223050 | 91.64% |
| FISH02BW | 79468943 | 11920341450 | 92.62% |
| FISH02BX | 70875549 | 10631332350 | 91.60% |
| FISH02BY | 79107561 | 11866134150 | 92.68% |
| FISH02BZ | 75266329 | 11289949350 | 92.62% |

## Figure S1: Venn diagrams of putative SNPs under selection for a) the complete dataset containing all sampling sites, and b) the dataset containing only New Zealand sampling sites.


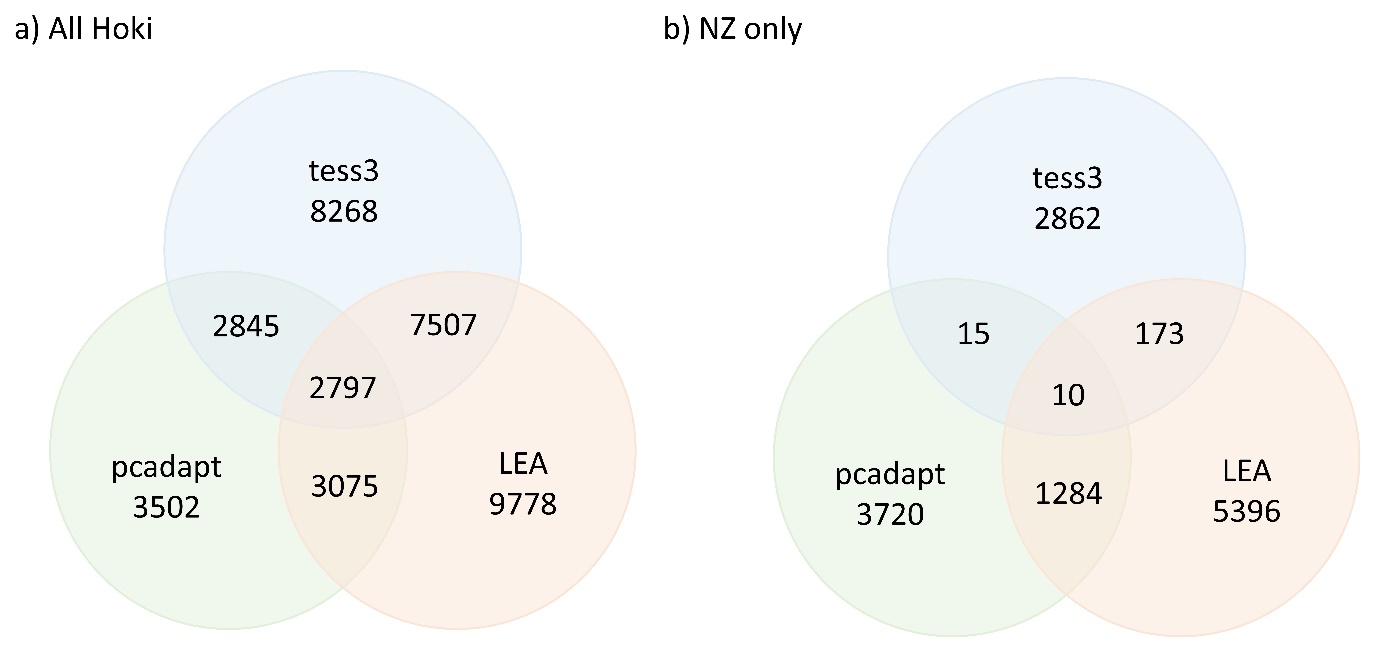


**Figure S2: Cross-validation plots for DAPC and LEA analyses for the Hoki dataset consisting of all locations and all SNPs. a) kmeans clustering analysis BIC values, versus the number of genetic clusters (K) tested, ranging from 1 to 30. K = 2 was determined to be the optimum K value. b) The number of principal components (PCs) vs a-score criterion tested prior to running DAPC analysis. The red circle indicates the optimum number of PCs = 71. c) Cross-entropy criterion values versus the number of factors tested in LEA snmf runs. K = 2 was determined to be the optimum K value.**

**
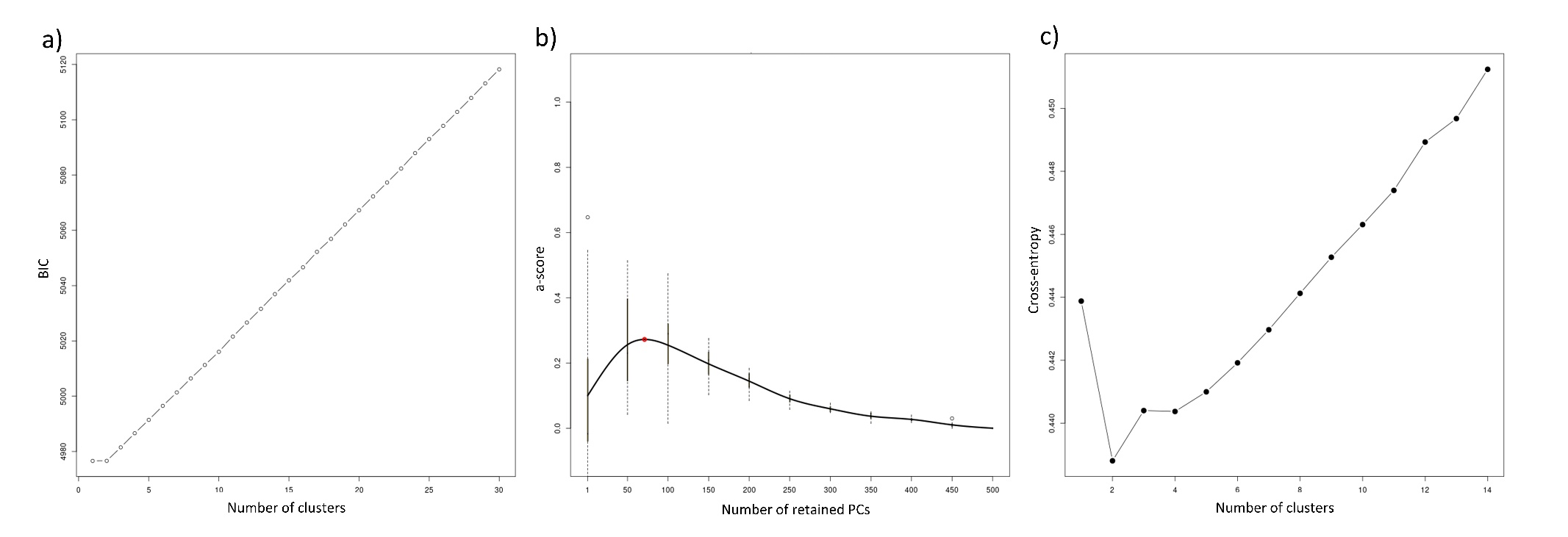
**

**Figure S3: Cross-validation plots for DAPC and LEA analyses for the Hoki dataset consisting of all SNPs and only New Zealand samples. a) kmeans clustering analysis BIC values, versus the number of genetic clusters (K) tested, ranging from 1 to 30. K = 1 was determined to be the optimum K value. b) The number of principal components (PCs) vs a-score criterion tested prior to running DAPC analysis. The red circle indicates the optimum number of PCs = 71. c) Cross-entropy criterion values versus the number of factors tested in LEA snmf runs. K = 1 was determined to be the optimum K value.**


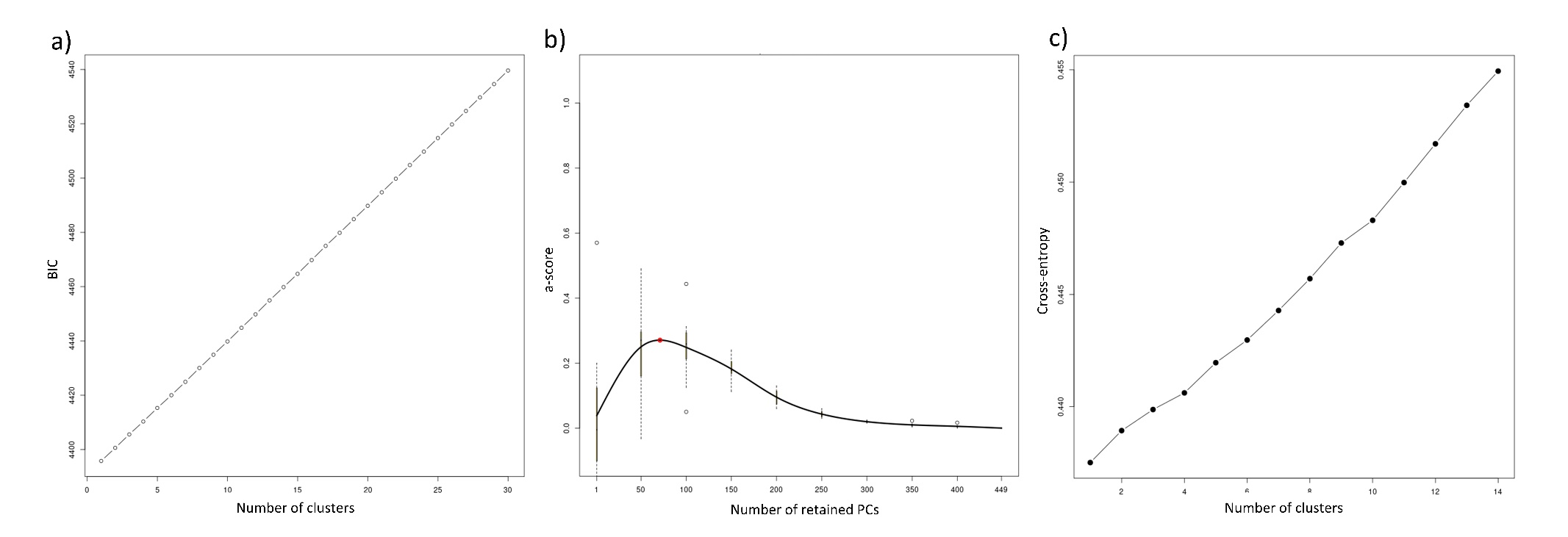


**Figure S4: Population genomic analysis outputs for the hoki dataset consisting of all SNPs and only New Zealand sampling sites: a) DAPC scatterplot of DA1 (34%) and DA2 (14%) with points coloured by sampling sites, b) pairwise Fst heatmap with hierarchical clustering dendrogram ― darker blues indicate higher pairwise Fst values and lighter blues indicate lower pairwise Fst values, and c) LEA ancestral admixture plots for K = 2 and K =** 3.


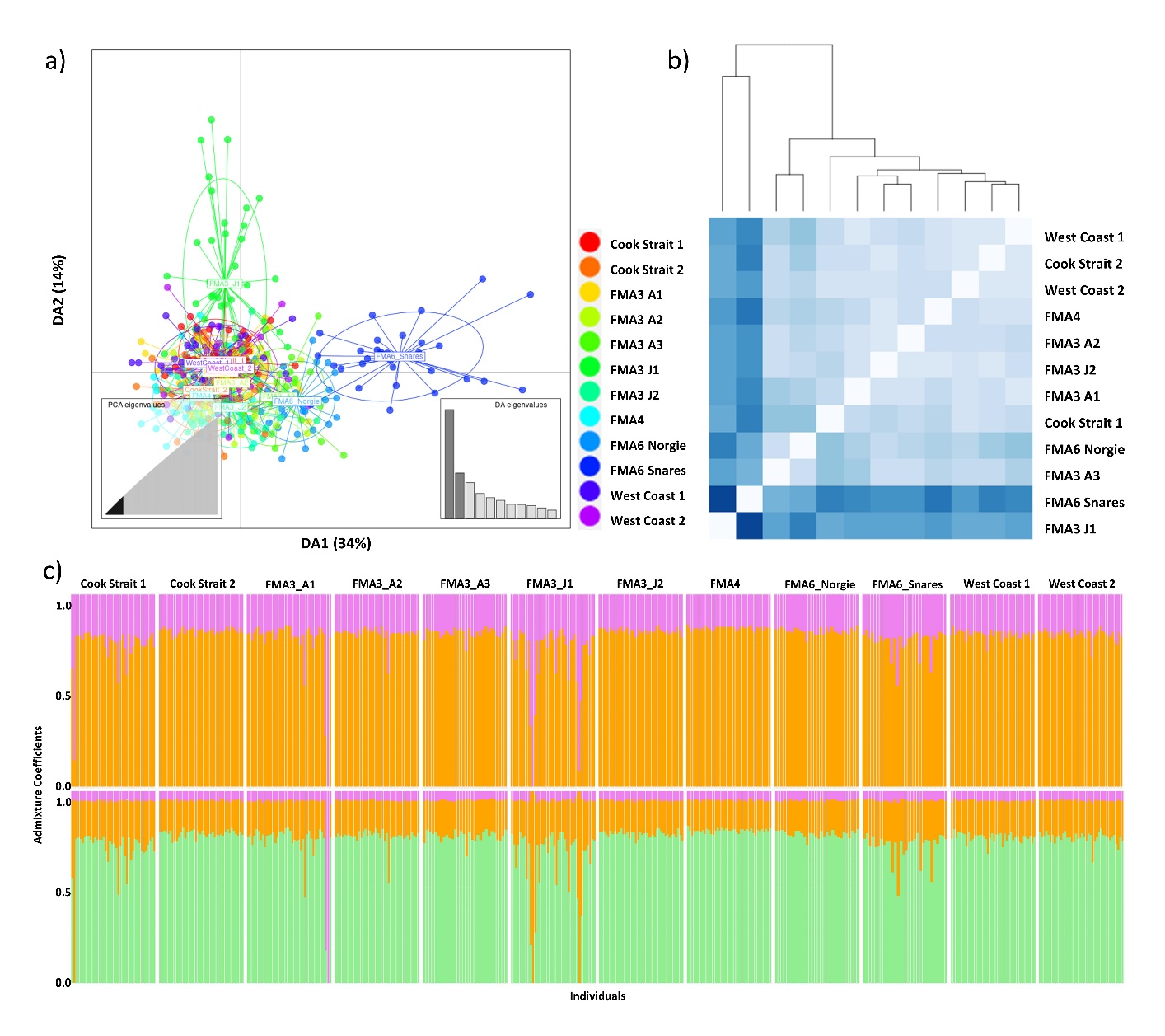


## Table S2: Diversity statistics for two of the hoki datasets: neutral SNPs and all sampling sites, and adaptive SNPs and all sampling sites. Π = nucleotide diversity, H_o_ = observed heterozygosity, H_e_ = expected heterozygosity, * = observed heterozygosity is higher than expected heterozygosity.

| **Neutral SNPs, all sampling sites** | **π (mean)** | **H_o_ (mean)** | **H_e_ (mean)** | **Tajima’s D (mean)** |
| --- | --- | --- | --- | --- |
| CookStrait_spawning_trawl1 | 0.171 | 0.191 | 0.169 | -0.317 |
| CookStrait_spawning_trawl2 | 0.167 | 0.179 | 0.165 | -0.360 |
| FMA3_adult_trawl1 | 0.170 | 0.185 | 0.167 | -0.334 |
| FMA3_adult_trawl2 | 0.167 | 0.181 | 0.165 | -0.353 |
| FMA3_adult_trawl3 | 0.168 | 0.180 | 0.165 | -0.417 |
| FMA3_juvenile_trawl1 | 0.171 | 0.191 | 0.169 | -0.307 |
| FMA3_juvenile_trawl2 | 0.167 | 0.179 | 0.165 | -0.359 |
| FMA4_adult | 0.166 | 0.176 | 0.164 | -0.371 |
| FMA6_adults_Norgie | 0.167 | 0.178 | 0.164 | -0.424 |
| FMA6_adults_Snares | 0.171 | 0.188 | 0.168 | -0.382 |
| Tasmania_adults1 | 0.195 | 0.249 | 0.191 | -0.095 |
| Tasmania_adults2 | 0.181 | 0.225 | 0.178 | -0.232 |
| WestCoast_spawning_trawl1 | 0.168 | 0.183 | 0.166 | -0.348 |
| WestCoast_spawning_trawl2 | 0.168 | 0.182 | 0.166 | -0.350 |
| **Adaptive SNPs, all sampling sites** | **π (mean)** | **H_o_ (mean)** | **H_e_(mean)** | **Tajima’s D (mean)** |
| CookStrait_spawning_trawl1 | 0.134 | 0.140 | 0.132 | -0.492 |
| CookStrait_spawning_trawl2 | 0.124 | 0.126 | 0.123 | -0.474 |
| FMA3_adult_trawl1 | 0.129 | 0.132 | 0.128 | -0.451 |
| FMA3_adult_trawl2 | 0.126 | 0.129 | 0.124 | -0.467 |
| FMA3_adult_trawl3 | 0.129 | 0.132 | 0.127 | -0.497 |
| FMA3_juvenile_trawl1 | 0.134 | 0.141 | 0.132 | -0.401 |
| FMA3_juvenile_trawl2 | 0.125 | 0.127 | 0.124 | -0.468 |
| FMA4_adult | 0.125 | 0.126 | 0.123 | -0.468 |
| FMA6_adults_Norgie | 0.126 | 0.128 | 0.124 | -0.522 |
| FMA6_adults_Snares | 0.132 | 0.137 | 0.130 | -0.472 |
| Tasmania_adults1 | 0.442 | 0.638 | 0.434 | 1.495 |
| Tasmania_adults2 | 0.419 | 0.570 | 0.412 | 1.341 |
| WestCoast_spawning_trawl1 | 0.128 | 0.131 | 0.126 | -0.460 |
| WestCoast_spawning_trawl2 | 0.129 | 0.130 | 0.127 | -0.450 |

**Figure S5: Cross-validation plots for DAPC and LEA analyses for the Hoki dataset consisting of neutral SNPs and all samples. a) kmeans clustering analysis BIC values, versus the number of genetic clusters (K) tested, ranging from 1 to 30. K = 2 was determined to be the optimum K value. b) The number of principal components (PCs) vs a-score criterion tested prior to running DAPC analysis. The red circle indicates the optimum number of PCs = 71. c) Cross-entropy criterion values versus the number of factors tested in LEA snmf runs. K = 2 was determined to be the optimum K value.**


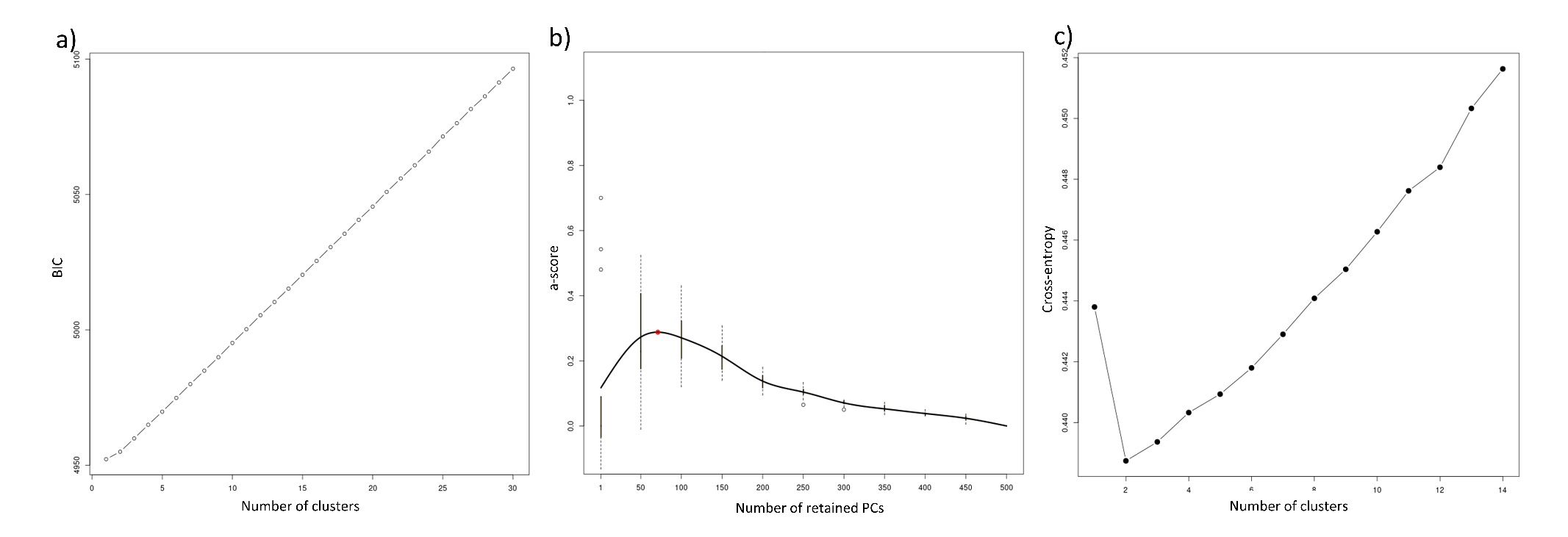


**Figure S6: Cross-validation plots for DAPC and LEA analyses for the Hoki dataset consisting of neutral SNPs and New Zealand only samples. a) kmeans clustering analysis BIC values, versus the number of genetic clusters (K) tested, ranging from 1 to 30. K = 1 was determined to be the optimum K value. b) The number of principal components (PCs) vs a-score criterion tested prior to running DAPC analysis. The red circle indicates the optimum number of PCs = 63. c) Cross-entropy criterion values versus the number of factors tested in LEA snmf runs. K = 1 was determined to be the optimum K value.**


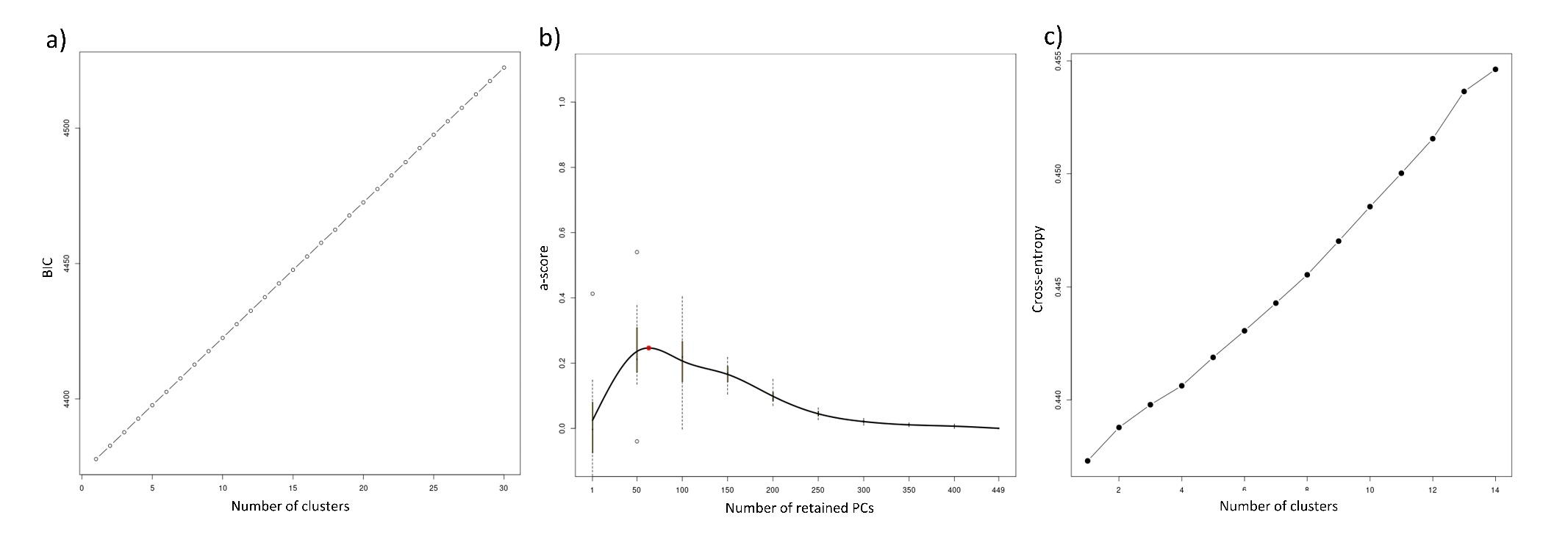


## Figure S7: Population genomic analysis outputs for the hoki dataset consisting of adaptive SNPs and all sampling sites: a) DAPC scatterplot of DA1 (34%) and DA2 (14%) with points coloured by sampling sites, b) pairwise Fst heatmap with hierarchical clustering dendrogram ― darker blues indicate higher pairwise Fst values and lighter blues indicate lower pairwise Fst values, and c) LEA ancestral admixture plots for K = 2 and K = 3.


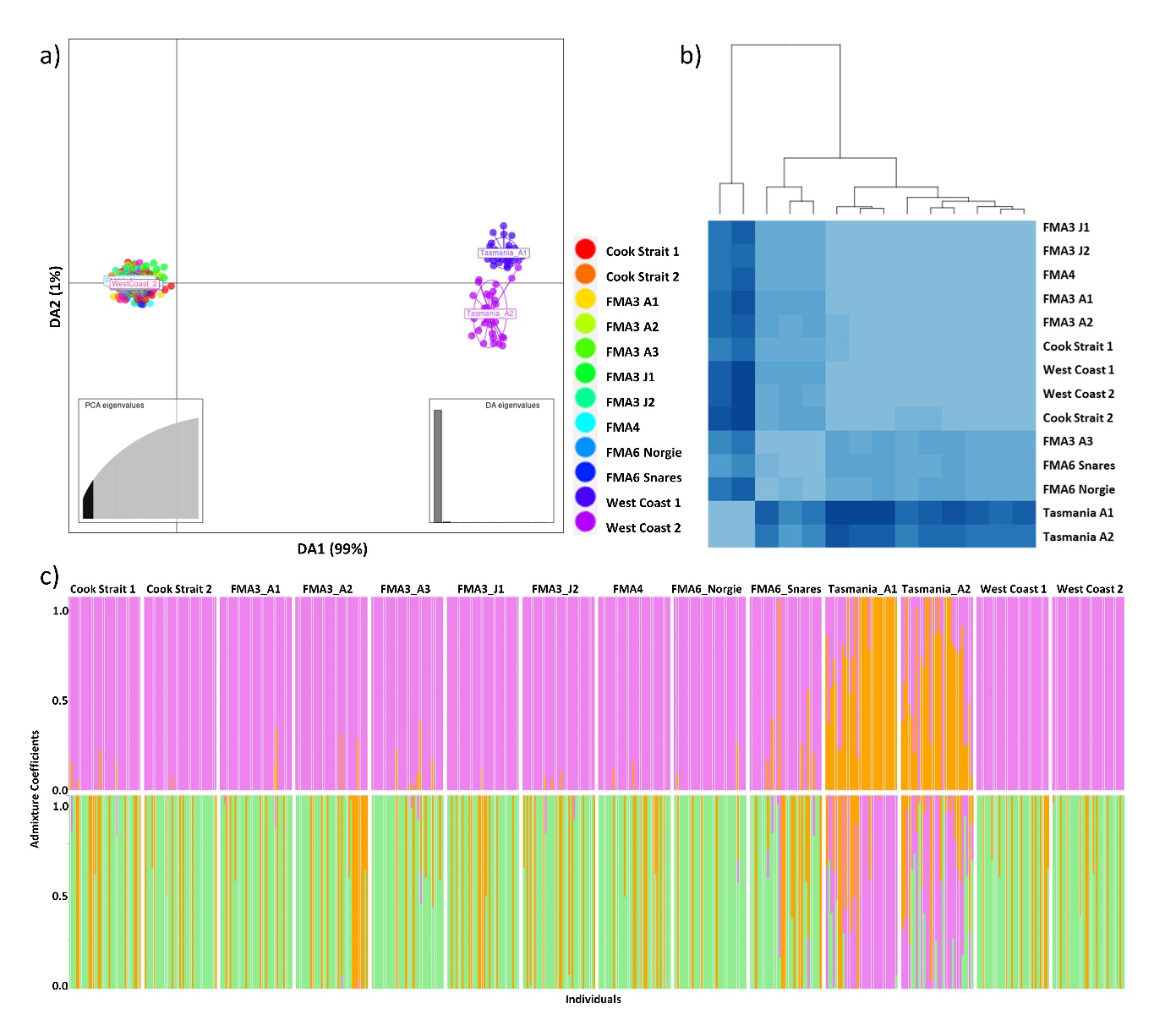


**Figure S8: Cross-validation plots for DAPC and LEA analyses for the Hoki dataset consisting of adaptive SNPs and all samples. a) kmeans clustering analysis BIC values, versus the number of genetic clusters (K) tested, ranging from 1 to 30. K = 2 was determined to be the optimum K value. b) The number of principal components (PCs) vs a-score criterion tested prior to running DAPC analysis. The red circle indicates the optimum number of PCs = 18. c) Cross-entropy criterion values versus the number of factors tested in LEA snmf runs. K = 2 was determined to be the optimum K value.**

**
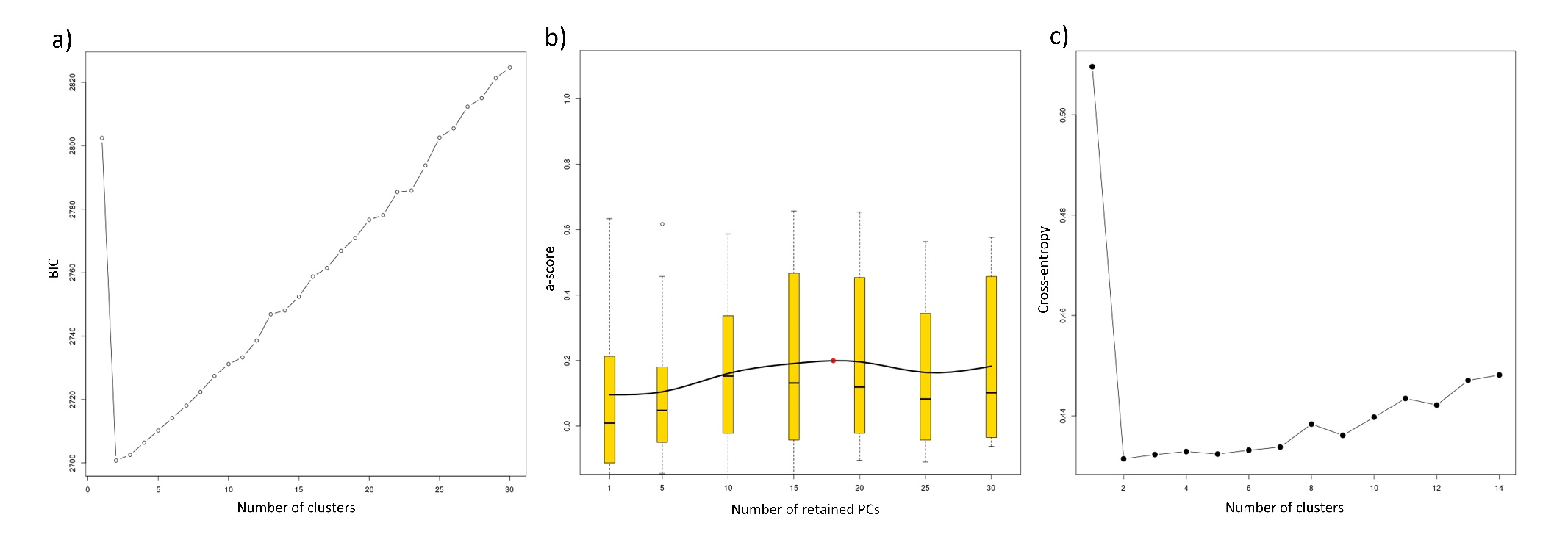
**

**Figure S9: Cross-validation plots for DAPC and LEA analyses for the Hoki dataset consisting of adaptive SNPs and New Zealand only samples. a) kmeans clustering analysis BIC values, versus the number of genetic clusters (K) tested, ranging from 1 to 30. K = 1 was determined to be the optimum K value. b) The number of principal components (PCs) vs a-score criterion tested prior to running DAPC analysis. The red circle indicates the optimum number of PCs = 7. c) Cross-entropy criterion values versus the number of factors tested in LEA snmf runs. K = 1 was determined to be the optimum K value.**

**
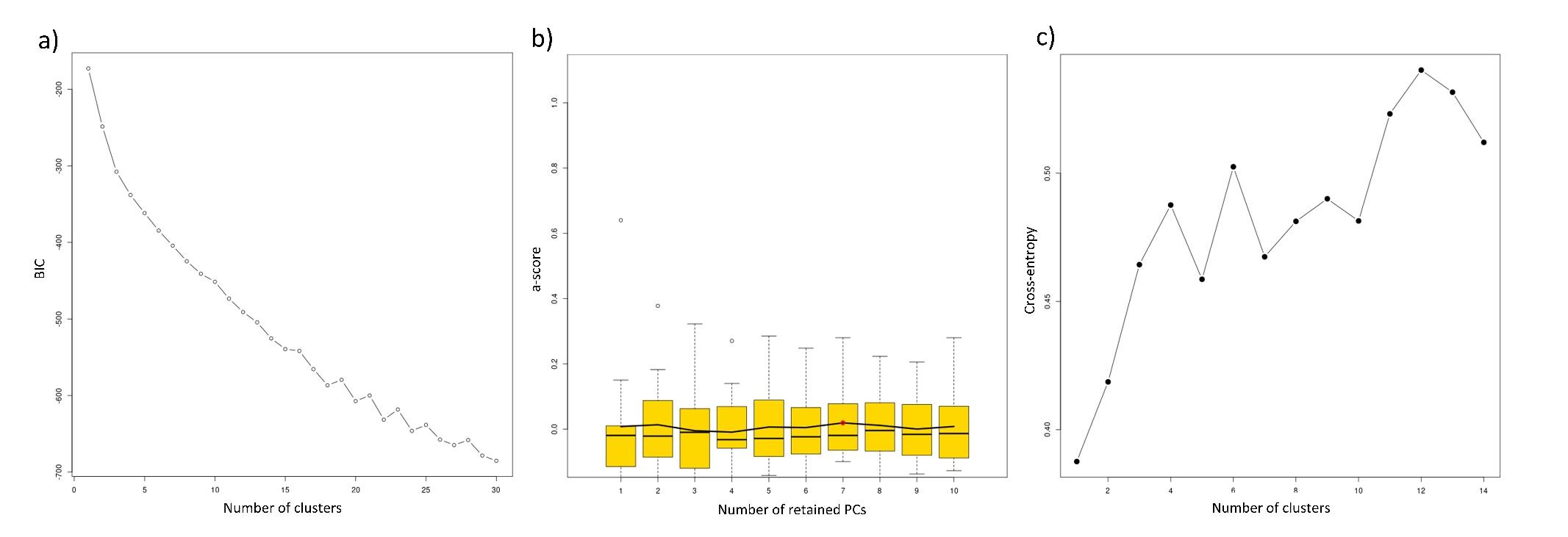
**

## Table S3: SNeP historical N_e_ output

| **GenAgo** | **N_e_** | **dist** | **r2** | **r2SD** | **items** |
| --- | --- | --- | --- | --- | --- |
| 189 | 42816 | 263206 | 0.002213 | 0.003146 | 2106 |
| 238 | 54208 | 209315 | 0.002198 | 0.003053 | 4066 |
| 299 | 65356 | 167042 | 0.002285 | 0.003191 | 9497 |
| 377 | 84516 | 132463 | 0.002228 | 0.003115 | 19380 |
| 462 | 102779 | 108040 | 0.002246 | 0.003227 | 25524 |
| 558 | 124581 | 89463 | 0.002238 | 0.003126 | 26830 |
| 666 | 148240 | 74984 | 0.002244 | 0.003183 | 34113 |
| 775 | 172884 | 64496 | 0.002237 | 0.003114 | 15921 |
| 828 | 184374 | 60346 | 0.002242 | 0.003208 | 24300 |
| 920 | 210286 | 54345 | 0.002183 | 0.003031 | 6750 |
| 962 | 212319 | 51946 | 0.002262 | 0.003213 | 11475 |
| 984 | 222407 | 50785 | 0.002208 | 0.003094 | 15290 |
| 994 | 224534 | 50256 | 0.002211 | 0.003106 | 4654 |
| 999 | 228681 | 50043 | 0.00218 | 0.002941 | 673 |

## Figure S10: Plot of historical N_e_ generated by SNeP


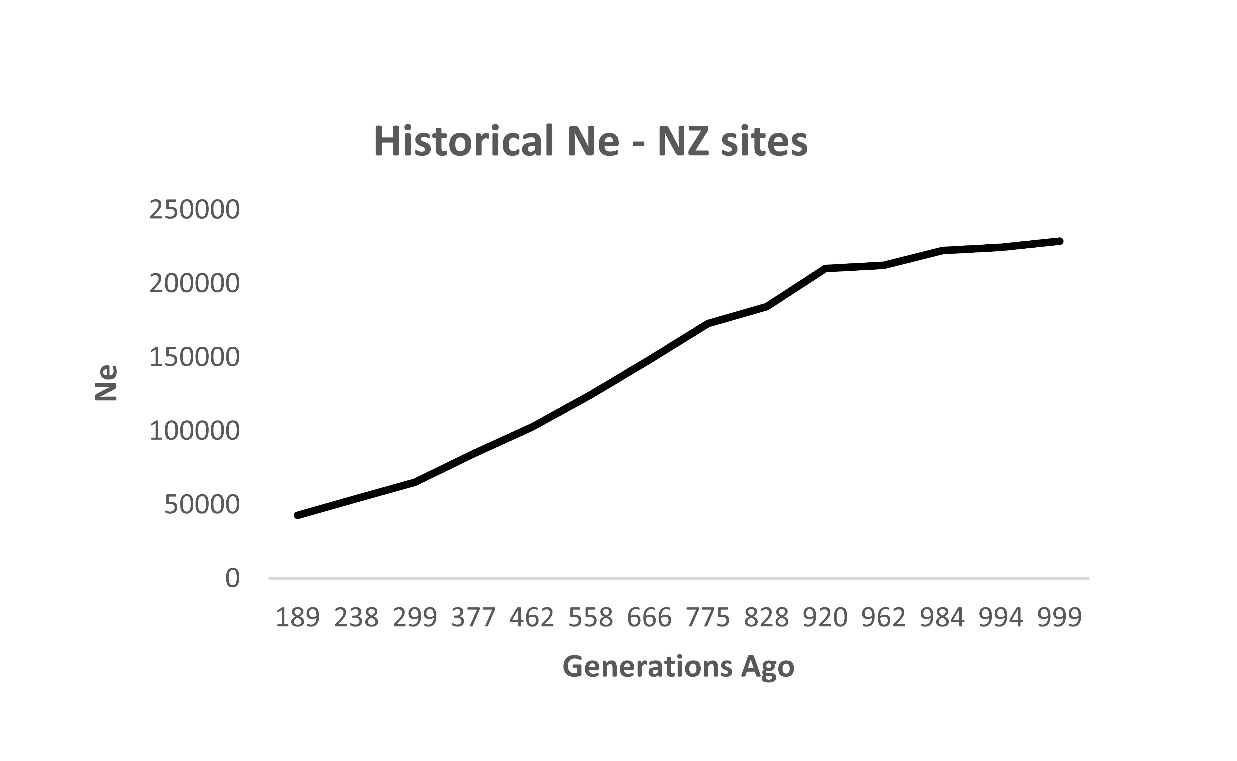

Supplement: Supplementary file 1 — Supplementary Material [file EVA-14-2848-s001.docx]
